# Genome sequences identify three families of Coleoptera as morphologically derived click beetles (Elateridae)

**DOI:** 10.1038/s41598-018-35328-0

**Published:** 2018-11-20

**Authors:** Dominik Kusy, Michal Motyka, Matej Bocek, Alfried P. Vogler, Ladislav Bocak

**Affiliations:** 1 Laboratory of Molecular Systematics, Department of Zoology, Faculty of Science, Palacky University, 17. listopadu 50, 771 46 Olomouc, Czech Republic; 2 Department of Life Science, Natural History Museum, Cromwell Road, London, SW7 5BD UK; 3 Department of Life Science, Silwood Park Campus, Imperial College London Ascot, London, SL5 7BD UK

## Abstract

Plastoceridae Crowson, 1972, Drilidae Blanchard, 1845 and Omalisidae Lacordaire, 1857 (Elateroidea) are families of the Coleoptera with obscure phylogenetic relationships and modified morphology showing neotenic traits such as soft bodies, reduced wing cases and larviform females. We shotgun sequenced genomes of *Plastocerus*, *Drilus* and *Omalisus* and incorporated them into data matrices of 66 and 4202 single-copy nuclear genes representing Elateroidea. Phylogenetic analyses indicate their terminal positions within the broadly defined well-sclerotized and fully metamorphosed Elateridae and thus Omalisidae should now be considered as Omalisinae stat. nov. in Elateridae Leach, 1815. The results support multiple independent origins of incomplete metamorphosis in Elateridae and indicate the parallel evolution of morphological and ecological traits. Unlike other neotenic elateroids derived from the supposedly pre-adapted aposematically coloured and unpalatable soft-bodied elateroids, such as fireflies (Lampyridae) and net-winged beetles (Lycidae), omalisids and drilids evolved from well-sclerotized click beetles. These findings suggest sudden morphological shifts through incomplete metamorphosis, with important implications for macroevolution, including reduced speciation rate and high extinction risk in unstable habitats. Precise phylogenetic placement is necessary for studies of the molecular mechanisms of ontogenetic shifts leading to profoundly changed morphology.

## Introduction

The elateroid beetles (Coleoptera: Elateroidea) are a morphologically heterogeneous group and include two different body plans: the well sclerotized ‘true elateroids’ (Elateridae, Eucnemidae, Throscidae), many of them able to jump for predator evasion using the long prosternal process fitting in the mesosternal cavity, and the soft-bodied ‘cantharoids’, some of them with larviform or incompletely metamorphosed females, all with short prosternum and unable to jump (Cantharidae, Lycidae, Lampyridae, Phengodidae, etc.). Taxonomists have accepted the paradigm of two reciprocally monophyletic groups corresponding to these types, the Cantharoidea and Elateroidea, until the late 20^th^ century when the cantharoid families were recognized as a sub-lineage within the more broadly defined Elateroidea^1,2^. Nevertheless, the concept of the ‘cantharoid’ clade was not questioned even after they were subsumed in the elateroids, on the assumption that soft-bodiedness originated only once^3–5^. When molecular data became available, multiple origins of soft-bodied elateroid families were proposed^6^. Nowadays, both the soft-bodied and the hard-bodied groups are known to be composed of distantly related families^7–11^.

The confusion about the relationships of soft-bodied groups also produced uncertainty for the placement of three small elateroid families, Omalisidae Lacordaire, 1857, Drilidae Blanchard, 1845 and Plastoceridae Crowson, 1972, which traditionally have been placed in the cantharoid clade^3,4^. In the latest morphological study, Omalisidae, a family of only about twenty known species were recovered as closely related to Lampyridae, Phengodidae and Telegeusidae (the fireflies and glow-worms)^5^, whereas molecular studies placed them in various positions either as a sister group to the hard-bodied Elateridae or as a terminal lineage within Elateridae, but in all cases very distant from Lampyridae and Telegeusidae^6,8,12^. Drilidae, the ‘false fireflies’, are a small group of predatory soft-bodied beetles. They have incompletely metamorphosed females with only head and appendages expressing the adult traits. Molecular data placed them in a derived position within Elateridae, specifically in the subfamily Agrypninae, as the tribe Drilini^7,13^. A recent analysis of basal coleopteran relationships that sampled both Drilidae and Omalisidae recovered them together with cardiophorine click beetles, but the conclusions were problematic for splitting the core groups of Elateridae^8^. The third ‘family’ under consideration here, Plastoceridae, represented by *Plastocerus* Schaum, 1852, have been firmly placed within the soft-bodied cantharoids ever since their formal recognition^1^. However, molecular data tentatively placed them within Elateridae near *Oxynopterus* Hope, 1842, leading to a proposed ranking as a subfamily of Elateridae^14^, in conflict with the earlier morphology-based analyses^4,5^. All molecular studies have been using only Sanger data, i.e., a low number of mitochondrial and rRNA markers. The molecular findings are counterintuitive and have been met with skepticism. Therefore, most taxonomists still assign Drilidae the status of a family and do not accept the results of the molecular studies and proposed changes in the formal classification^5,8,15–18^.

Among soft-bodied groups, several lineages exhibit larviform or incompletely metamorphosed females, which also occur in a few other beetles, e.g., *Thylodrias* Motschulsky, 1839, *Micromalthus*, Leconte, 1878 and *Ozopemon* Hagedorn, 1910, but they are most prevalent in Elateroidea^6,19–24^. The distinct appearance generally led to their taxonomic recognition at family rank^1,5,15^. Only females show the most extreme cases of larviform or partially metamorphosed morphology, although some traits may appear also in males^25,26^. The point at which metamorphosis is prematurely terminated across elateroid lineages is variable. The neotenic females of net-winged beetles (Lycidae), glow-worm beetles (Rhagophthalmidae) and some genera of fireflies (Lampyridae) are completely larviform, except for the fully developed reproductive organs. Various fireflies have only an adult-like pronotum and head, in drilids only the head and appendages are adult-like, omalisids exhibit a larviform abdomen, modified thorax and short elytra, and modifications in plastocerids are limited to the free abdominal sternites and lack the click mechanism^14,27–34^. The neotenics differ not only in their morphology, but also in ecological traits, which affect macroevolution: the lineages with brachelytrous, wingless or completely larviform females occupy small ranges due to the low ability to disperse, they usually move only slowly, and are often unpalatable and aposematically coloured. Due to limited dispersal capacity, they are mostly limited to environmentally stable habitats and regions where populations persist with low risk of extinction. The lineages exhibiting neotenic females usually represent only a fraction of the species diversity compared to their fully winged sister groups. Most neotenics are uncommon and, therefore, often only males are known while the females are rarely seen or may not be known at all, but inferred to be neotenic based on certain modifications in the males^1,7,19,20,34^. Taxa with incompletely metamorphosed females have generally been found to be related with soft-bodied lineages. Their characteristic traits, such as low dispersal capacity, restricted ranges and chemical protection as an alternative anti-predatory strategy, have been considered as pre-adaptations which increase the profitability of the shift to incomplete metamorphosis and higher investment in offspring^19,20^.

The aim of this study is to produce genomic data for three enigmatic neotenic and morphologically aberrant lineages, Omalisidae, Drilidae and Plastoceridae and use them to investigate their relationships to other elateroid families. As the previous studies provided ambiguous phylogenetic signal, whole genome data are the ultimate source of information which could shed light on their phylogenetic relationships. These elateroids are unique by their divergent morphology and relictual distribution. Their robust placement is crucial for future studies on the molecular mechanisms of incomplete metamorphosis leading to weakly sclerotized bodies and winglessness.

## Results

**Figure 1:**
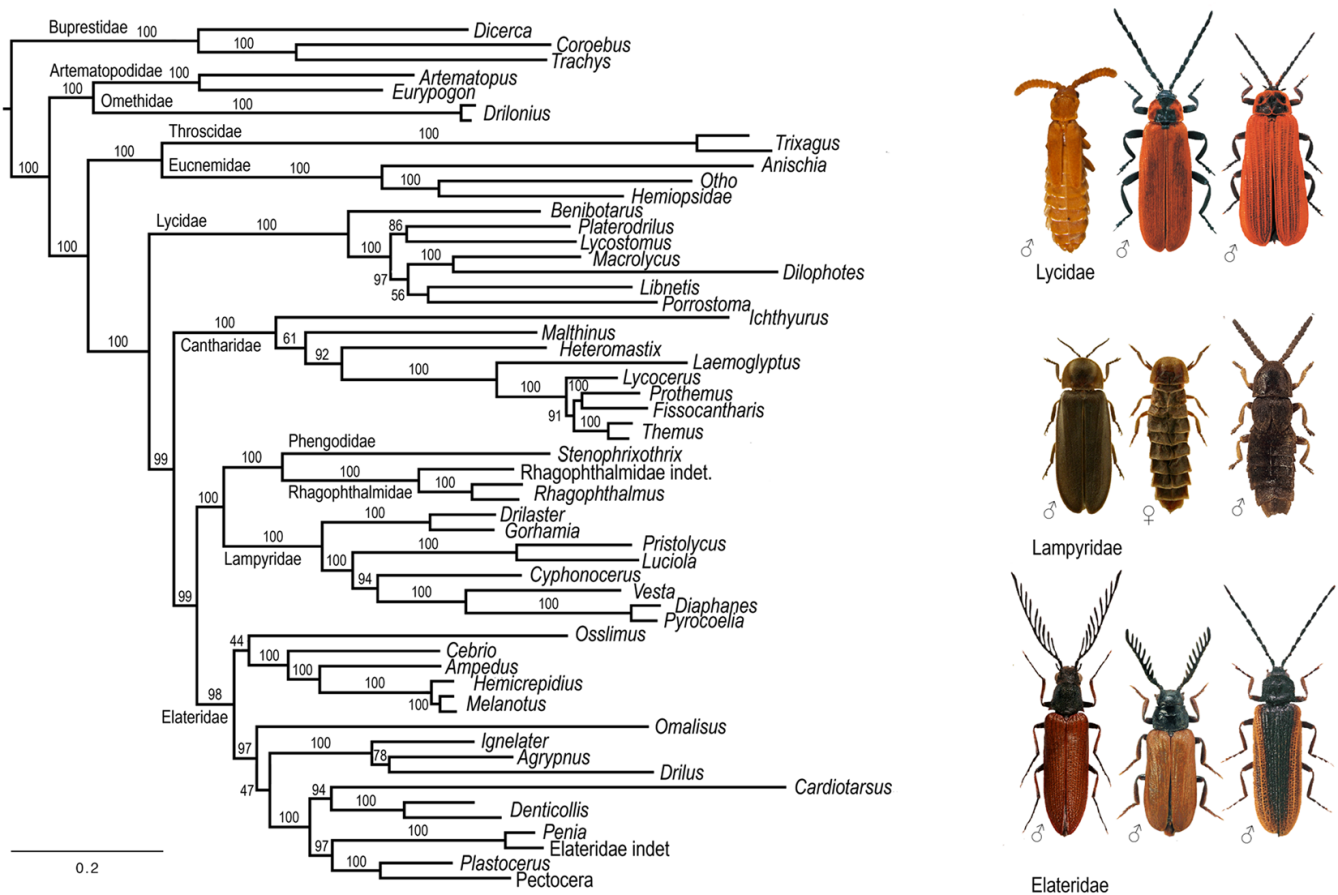
The maximum likelihood tree recovered from the 66-single copy protein coding genes at nucleotide level. Photographs of general appearance © authors.

The Fig. 1 shows the ML tree topology inferred from the 66-gene analysis at nucleotide level. The three focal taxa were found within Elateridae: *Plastocerus* associated with *Pectocera* (Oxynopterinae), *Drilus* associated with *Agrypnus* (Agrypninae) and *Omalisus* as a sister to the latter clades combined. The AU tests rejected alternative topologies (Table 1).

**Table 1:** Approximately unbiased test of alternative relationships of *Drilus*, *Omalisus*, and *Plastocerus* recovered by the analysis of the 66-taxa dataset.

Topology	logL	deltaL	p-AU
ML topology as in Fig. 1	−1035875	0.000	0.7247
Constraint:			
*Omalisus* + *Plastocerus* + *Drilus*			
sister to Elateridae	−1038925	3046.7	0.0001
*Drilus* sister to Cantharidae	−1037003	1124.9	0.0000
*Drilus* sister to Elateridae	−1036926	1048.2	0.0000
*Drilus* sister to Lampyridae	−1037133	1254.5	0.0000
*Drilus* sister to Lycidae	−1037070	1192.1	0.0000
*Omalisus* sister to Cantharidae	−1035963	84.5	0.0114
*Omalisus* sister to Elateridae	−1035942	63.8	0.0045
*Omalisus* sister to Lampyridae	−1036076	197.6	0.0000
*Omalisus* sister to Lycidae	−1036033	154.6	0.0004
*Plastocerus* sister to Cantharidae.	−1038735	2857.3	0.0000
*Plastocerus* sister to Elateridae	−1037933	2054.8	0.0006
*Plastocerus* sister to Lampyridae	−1038902	3023.6	0.0000
*Plastocerus* sister to Lycidae	−1038975	3096.6	0.0000

**Figure 2:**
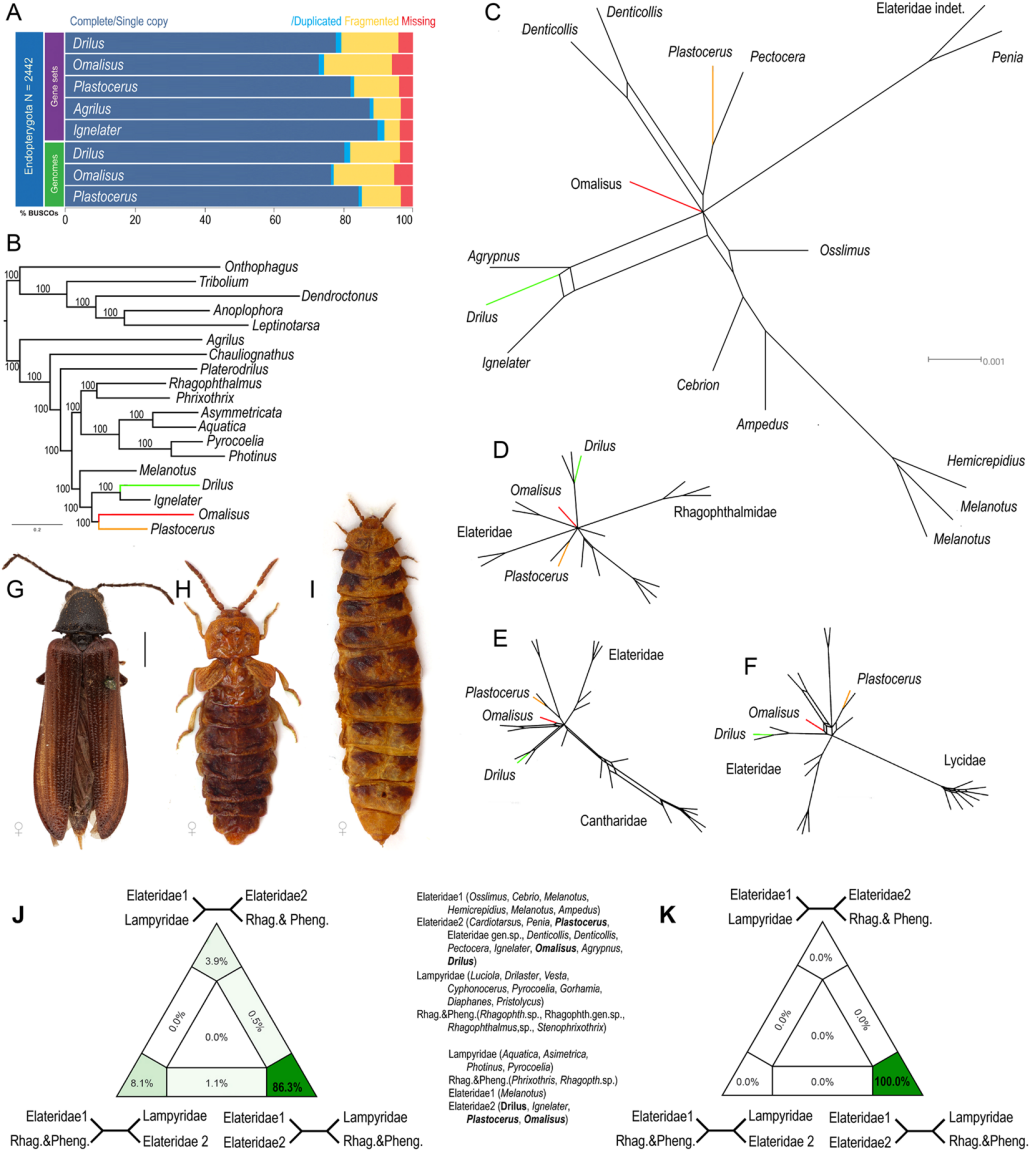
(**A**) Summarized benchmarks in the BUSCO assessment among assembled draft genomes and annotated gene sets. These estimations used 2442 expected Endopterygota genes as query; (**B**) Maximum likelihood tree obtained from the analysis of the 4202 ortholog dataset at nucleotide level; (**C**) Network obtained from the separate maximum likelihood analyses of Elateridae and all 66 single copy genes gene trees, (**D**) ditto, Elateridae and Rhagophthalmidae, (**E**) ditto, Elateridae and Cantharidae; (**F**) ditto, Elateridae and Lycidae; (**G**–**I**) General appearance of females: (**G**) *Plastocerus angulosus*, (**H**) *Omalisus fontisbellaquei*, (**I**) *Drilus flavescens*. Note the similar degree of morphological modifications in the males of some Lycidae and Lampyridae in Fig. 1. (**J**–**K**) 2D simplex graph. The support values in cells show support for each of the three topologies illustrated. (**J**) Topologies recovered using 66-taxa dataset as nucleotide level; (**K**) Topologies recovered using genomic dataset at nucleotide level. Photographs of general appearance of females © authors.

Newly generated shotgun genomic sequencing data provided high coverage of genomes at a sequencing depth of 69–312×. Data were used to create an ortholog set of 4202 genes from publicly available transcriptome and genome data of Coleoptera. The genome size was estimated to ~536 million base pairs (Mbp) for *D. mauritanicus*, 367 Mbp for *P. angulosus* and 270 Mbp for *O. fontisbellaquei* (Fig. S1). The genome completeness is summarized in Fig. 2A and the completeness of datasets in Figs S2–S7. The dataset based on 4202 orthologs recovers an almost identical topology at amino acid and nucleotide levels or filtering approach (Figs S8–S14). Taking into account the lower taxon sampling, *Omalisus* was recovered as sister to *Plastocerus*, unlike in the 66-gene dataset which placed it as sister to a wider clade that also includes *Drilus*, but with poor support. The genomic dataset includes only two elaterids, *Melanotus* and *Ignelater*, but they represent the two major clades of the family (Fig. 2). Because of the early branching of *Melanotus*, the three neotenic lineages are safely placed within the Elateridae (Fig. 2), with 100% support at all nodes.

Further exploration of the 66-gene dataset based on individual gene trees from individual loci and supernetwork analysis, using various subsets of taxa in addition to the Elateridae, always grouped the three focal groups within Elateridae, in positions very compatible with the supermatrix approach on the full taxon set (Fig. 2C–F). The Four cluster Likelihood Mapping (FcLM) analysis identified predominant support for the monophyly of all Elateridae including the three focal taxa and Rhagophthalmidae/Phengodidae + Lampyridae as a sister to them (Fig. 2J,K). Further ML topologies recovered from 66-taxa dataset, the coalescent trees recovered from both datasets and the tests of the alternative relationships are shown in Figs S15–S23.

## Discussion

The current study is based on the most extensive transcriptomic dataset of ‘cantharoid’ families to date. At the base of the tree, the results recover three ‘cantharoid’ lineages, i.e. Cantharidae, Lycidae and the bioluminescent clade, composed of the Lampyridae, Rhagophthalmidae, and Phengodidae. The monophyly of the latter is in contrast to some studies that separated these three families and suggested that bioluminescence in adult Lampyridae has a separate evolutionary origin from the other two families with exclusively larval bioluminescence^6,7^. It seems now unlikely and the current topology is in agreement with eight-gene analysis^8^ and with morphology^4^. The widely defined Elateridae, including the three small ‘cantharoid’ lineages Drilidae, Omalisidae and Plastoceridae is the sister of the bioluminescent clade (Figs 1 and 2). The families traditionally assigned to the morphology-based Cantharoidea^1^ acquired soft-bodiedness independently, or alternatively reverted to hard-bodied forms in the Elateridae (Fig. 1). Whatever the ancestral state, neotenic lineages may be derived both from soft-bodied and hard-bodied ancestors.

The morphology-based classifications have emphasized morphological divergence of Omalisidae, Drilidae, and Plastoceridae, and thus assigned them family rank^4,5,8,15–18^ although the possible relationships of *Omalisus* and Elateridae was mentioned already in the 19^th^ century^35^. These taxa share some characters with the ‘cantharoid’ soft-bodied elateroids (Fig. 2H,I) and even if not all traits are present^14^, morphological phylogenetic analyses have never found the proximity of these taxa to the well-sclerotized click beetles^4,5^.

In contrast with morphology, molecular analyses indicate that phenotypically similar elateroids are not necessarily closely related. Initial hints of cantharoid non-monophyly^6,12^ were corroborated later^7,8,10^. Kundrata & Bocak^13^ were the first to hypothesize that the unrelated forms exhibiting incompletely metamorphosed females might have originated also within click beetles and proposed that Drilidae are a terminal branch in the elaterid subfamily Agrypninae, consistent with the tribal level (Drilini). These results have been treated as conjectural or have been ignored^5,8,15–18^. Recently, also based on the rRNA and mtDNA markers, the Plastoceridae was down-ranked to a subfamily in Elateridae and inferred as a sister lineage of *Oxynopterus* (Oxynopterinae)^14^, but also with only moderate support due to a limited amount of information. All earlier studies have been based on Sanger-era markers and some lacked adequate taxon sampling. Therefore, a robust placement was not obtained^6–9,13,36,37^. Similarly, the analysis of eight nuclear markers did not recover click beetles as a monophylum and no conclusive hypothesis could be proposed^8^. The rRNA-based analyses of Elateroidea produced ambiguous alignments when numerous length-variable sequences were combined in a single dataset which contained a broad set of Coleoptera, mis-aligning the comparatively short loops in the rRNA genes of elaterids^6^. Similarly, low genetic divergence is characteristic for the protein coding nuclear genes of Elateridae, which might have contributed to the failure to recover click beetle monophyly in the recent analysis of Zhang *et al*.^10^. Therefore, more conclusive evidence was sought in the genomic datasets. We investigate two contradicting phylogenetic hypotheses: (1) drilids, plastocerids and omalisids evolved within the elaterid clade, hence, they are in fact modified click beetles or (2) these lineages are deeply nested in Elateroidea and should be designated as families, as is still widely held.

The current study, based on tens of thousands nucleotide positions in the densely sampled 66-gene Elateroidea dataset and millions of positions in the genomic dataset, now confirms the placement of former Omalisidae, Drilidae and Plastoceridae in Elateridae with high support (Figs 1 and 2) and under a wide array of analytical approaches (Figs S8–S20) which were performed to test their impact on the resulting topology^38^. The topologies set *Drilus* and *Plastocerus* near *Agrypnus* and *Pectocera*, respectively, corroborating the earlier studies^13,14^, and *Omalisus* is robustly recovered as a deeply rooted branch in Elateridae, although in variable positions (Figs 1 and 2). Deep splits^11^ and rapid radiations^39,40^ often represent extremely difficult phylogenetic problems even when large datasets are analyzed (here, the unstable position of *Omalisus* or a conflicting signal for *Melanotus*; Figs S11–S21). We found that although the genomic and 66-gene datasets place *Omalisus* in Elateridae, the later cannot robustly identify its sister clade within this family (Figs 2, S15–S17, S20). Therefore, we studied in detail alternative positions of focal taxa and we found that the alternative hypotheses that force well-sclerotized elaterids to be monophyletic, or *Drilus*, *Omalisus*, and *Plastocerus* to be a sister to either Lampyridae, Cantharidae, Lycidae or Elateridae are all rejected by AU tests (Table 1). Similarly, the monophyly of all Elateridae including three focal families got predominant support (Fig. 2J,K). To sum up, it becomes difficult to dispute the possibility that the lineages with modified morphology (*Plastocerus*) and even with incompletely metamorphosed females (*Drilus*, *Omalisus*) are nested within the Elateridae. Thus, these taxa do not deserve the family rank despite their morphological uniqueness. Based on the current analysis, we propose to lower Omalisidae from the family rank to a subfamily Omalisinae Lacordaire, 1857 in Elateridae and we confirm the status of Drilini and Plastocerinae^13,14^.

The shift to incompletely metamorphosed females (Fig. 2H,I) is known to affect the macroevolution of a lineage^19^. The well-sclerotized elaterids contain the substantial part of the extant Elateroidea diversity, i.e. ~10,000 species^37^, and the morphologically modified elaterids are represented only by Plastocerinae (2 sp), Omalisinae (22 spp) and Drilini (>100 spp). Their evolutionary trajectory is determined by low species numbers, limited geographic ranges, and small population sizes^14,34,41,42^. Most omalisids and *Plastocerus angulosus* occur in the Mediterranean only and their ranges are usually restricted to costal refugia where broad leaf forests persisted during the last glacial maximum, indicating the constraints to their subsequent dispersal^14,34^. Drilini are widespread in the Afrotropical region, the Mediterranean and along the South Asian coast to Thailand, but similarly to omalisids they are known to have limited species ranges and are generally rare in ecosystems^41^. Hence, omalisids, drilids and plastocerids represent examples of lineages which diverted from a successful body-plan of click beetles whose morphology did not substantially change since the Late Triassic^43^ and which has been reproduced in thousands of extant species. Yet, they survive for a long time in stable ecosystems similarly to other neotenic elateroids^19,32,33^.

The secure placement of these three lineages within the true click beetles has important implications for the origin of neoteny and macroevolution of modified lineages. First, the derived positions within Elateridae demonstrates that astonishing differences in morphology can be achieved through what appears to be shifts in ontogenetic programs. The general appearance and individual morphological traits changed over short evolutionary time scales, and these differences lead to convergent morphologies observed in several unrelated groups throughout the Elateroidea. Second, these morphological shifts also have profound macroevolutionary consequences. The traditional placement suggested relationships of Elateridae, Eucnemidae and Throscidae^2^, and Elateridae became the dominant part of Elateroidea diversity with >40% of species, world-wide distribution and high dominance in many beetle communities since the Jurassic^43^. Strong body sclerotization and an effective escape mechanism apparently proved to be an evolutionarily successful design, to make Elateridae an example of a morphologically conservative lineage whose great species diversity was not accompanied by morphological diversity^43,44^. Yet, among these conservative groups some lineages arose which were very different morphologically and resemble distant neotenic relatives in Lycidae, Lampyridae and Rhagophthalmidae. It has generally been assumed that soft-bodiedness in Elateroidea might be the first symptom of unfinished metamorphosis^19,30^ raising the possibility for the more fully neotenic lineages raised within them. Soft-bodied elateroids move slowly, are commonly unpalatable^45,46^ and aposematically coloured^47^. These factors might increase the trade-off gains if dispersal capacity is further lowered in favor of higher fecundity due to incomplete metamorphosis^20^. Neotenics nested in click beetles now falsify the general validity of this hypothesis about ecological pre-adaptations required for the origin of arrested or prematurely terminated metamorphosis^20^. If there are life-history pre-adaptations for incomplete metamorphosis present in Elateroidea, they differ in individual groups and might not have only the ecological character. Based on the origins of neoteny in click beetles, the alternative hypothesis can be formulated: a rapid modification of the regulatory system of insect metamorphosis at molecular level^48,49^ might be a trigger for a subsequent improvement by the natural selection, such as large-bodied females, higher fecundity and evolution of alternative defensive strategies. To sum up, we can say that convergent evolution has produced multiple lineages that ‘replay life’s tape’^50,51^ exploring a life strategy that is favored by stable climatic and ecological conditions, and which can apparently start from different ancestral states. As this shift is triggered by prematurely arrested metamorphosis, the repeated origin may indicate the low stability of the molecular regulatory system in Elateroidea. The identification of the closest relatives of neotenics may therefore be the first step for a comparative biology of the mechanisms of metamorphosis.

## Methods

### Material, laboratory procedures, and draft genomes

Total genomic DNA was extracted from single adult specimens of *Omalisus fontisbellaquei* Geoffroy, 1785 (from Czechia), *Drilus mauritanicus* Lucas, 1842 (Spain) and *Plastocerus angulosus* Schaum, 1852 (Turkey) using the DNeasy kit (Qiagene Inc., Hilden, Germany). The voucher specimens have been deposited in the collection of Department of Zoology, Palacky University, Olomouc. Genomic DNA of all three specimens was shotgun sequenced on the Illumina X Ten platform (Illumina Inc., San Diego, CA) for 2 × 150 bp paired-end reads by Novogene Co., Ltd. (Beijing, China) and each individual was sequenced for 30–107 × 10^9^ base pairs. Raw paired-end reads were filtered using fastp 0.13.2^52^ under the following parameters -q 5 -u 50 -l 50 -n 15 and other settings as default. The filtering steps included the removal of read pairs if either one read contains adapter contamination; if the proportion of low quality bases is over 50%; or if either one read contains more than 15 N bases. The quality of reads was visualized with FastQC (http://www.bioinformatics. babraham.ac.uk/projects/fastqc). Sequence data were deposited in GenBank SRA (Accession Numbers AB123456-AB123456).

We performed k-mer counts on the filtered data in Jellyfish 2.2.7^53^ using 31-mer sizes. Moreover, based on the distribution of k-mer occurrences, we estimated the genome size using GenomeScope^54^ and assembled draft genomes of *O. fontisbellaquei*, *D. mauritanicus* and *P. angulosus* using MEGAHIT 1.1.3^55,56^, with all parameters set to default and k-mer sizes of 31, 59, 87, 115 and 143. Additionally, the genome of *O. fontisbellaquei* was processed using the Shovill pipeline (https://github.com/tseemann/shovill) and assembled with SPAdes 3.12.0^57^ using k-mer size 31, 51, 71, 91 and 111 under default parameters. We produced statistics of draft genomes with the assembly-stats algorithm (https://github.com/rjchallis/assembly-stats) and the results of both methods used for *O. fontisbellaquei* were similar and are shown in Fig. S24. The SPAdes contigs were used for further analyses of *Omalisus*. Obtained contig sequences were used to train Augustus^58^ for species specific gene models with BUSCO 3^59^, -long option, Endopterygota set of conserved genes (n = 2442) and -sp tribolium2012 as the closest relative. Predicted species specific gene models were then used for *ab initio* gene predictions in Augustus, and predicted protein coding sequences were used for subsequent analyses in Orthograph 0.6.1^60^. The genome and coding gene set completeness was evaluated based on the predicted protein sets with BUSCO using expected 2442 Endopterygota single-copy orthologs as targets. BUSCO quantitatively assesses completeness using evolutionary conserved expectations of gene content. We compared completeness of predicted protein sets with *Agrilus planipennis* and *Ignelater luminosus*.

### Data matrices

Two datasets were assembled for the phylogenetic analysis:

(1) Single-copy genes – 66-taxa dataset (Table S1). This dataset is based on PCR amplified sequences for 95 genes across Coleoptera^10^, of which 66 gene for 54 taxa were retained after the removal of 29 supposedly multi-copy genes, as described earlier^11^. The putative homologs for *O. fontisbellaquei*, *D. mauritanicus* and *P. angulosus* were added to the earlier published data. Exons were concatenated to produce a supermatrix of 53,253 aligned positions.

(2) Genome orthologs. Transcriptomes of *Melanotus cribricollis*^61^; *Asymmetricata circumdata, Aquatica ficta, Pyrocoelia pectoralis*, *Rhagophthalmus* sp^62^; *Chauliognathus flavipes* and *Phrixothrix hirtus*^63^ were downloaded from the NCBI SRA archive and assembled as described by Kusy *et al*.^11^. Additionally, we downloaded the gene set of *I. luminosus* from fireflybase.org^64^ and the transcriptome of *Photinus pyralis*^65^ from NCBI Transcriptome Shotgun Assembly database (Table S2). The ortholog set was obtained by searching the OrthoDB 9.1 database^66^ for one-to-one orthologs among Coleoptera in available genome sequences of *A. planipennis*^67^, *Anoplophora glabripennis*^68^, *Dendroctonus ponderosae*^69^, *Leptinotarsa decemlineata*^67^, *Onthophagus taurus*^67^, and *Tribolium castaneum*^70,71^ (Tables S3, S4). OrthoDB 9.1 specified 4225 protein coding single copy genes for the above species and the Coleoptera reference node. We used Orthograph 0.6.1^60^ to search the above transcriptomes and predicted protein coding gene sets for the corresponding sequences. Default settings were used. We summarized Orthograph data reporting 4202 orthologs, removed terminal stop codons and masked internal stop codons at the translational level and nucleotide levels using the perl script summarize_orthograph_results.pl^60^. The amino acid sequences were aligned using MAFFT 7.394 with the *L-INS-i* algorithm^72^. Resulting alignments from each ortholog group were checked for the presence of outliers using the script https://github.com/mptrsen/scripts/blob/master/outlier_check.pl and following the methods reported by Misof *et al*.^73^ and Peters *et al*.^74^. We used Pal2Nal^75^ to generate multiple sequence alignments of nucleotides corresponding to amino acids. Aliscore 2.0^76,77^ with the maximal number of pairwise comparisons, -e option and default settings were used to identify random or ambiguous similarity within alignments which were masked using Alicut 2.3 (https://github.com/mptrsen/scripts/blob/master/ALICUT_V2.3.pl) and Alinuc.pl^73^ to apply Aliscore results to the nucleotide data. The dataset of 4202 orthologs on amino acid and nucleotide levels were concatenated into supermatrices 1 and 2 (1–amino acids; 2–all nucleotides) with FASconCAT-G^78^. Additional filtered datasets were used for tree construction: (a) nucleotides, 1st + 2nd codon positions only; (b) amino acid raw data (no filtering e.g. outliers removal, Aliscore); (c) nucleotide raw data; (d) a subset of Supermatrix 1, orthologs available for all taxa; (e) a subset of Supermatrix 2 containing orthologs for all taxa. AliStat 1.7 (https://github.com/thomaskf/AliStat) was used to generate distributions of missing data per site in the supermatrices.

### Phylogenetic analyses

IQ-TREE 1.6.6^79^ and RaxML^80^ were used to calculate maximum likelihood (ML) trees, with partitions identified by the Model Finder tool of IQ-TREE and using the Bayesian Information Criterion^81,82^. The partitions, models and parameters are available upon request. The ultrafast bootstrap option was used with 3000 bootstrap iterations^83^. The IQ-TREE analyses were run with the -spp parameter allowing each partition to have its own evolutionary rate.

To investigate alternative and/or confounding signal in the 66-taxa dataset and genomic dataset, we used FcLM analysis^73,84^ implemented in IQ-TREE to study a possibility of the occurrence of incongruent signal in phylogenomic datasets that might not be revealed by a phylogenetic multi-species tree. Additionally, gene tree incongruence in 66 genes dataset was tested by visualizations of the dominant bipartitions among individual loci based on the individual IQ-TREE ML gene topologies by constructing supernetworks using the SuperQ method implemented in Spectre selecting the ‘balanced’ edge-weight with ‘JOptimizer’ optimization function, and applying no filter^85,86^. This methodology decomposes all gene trees into quartets to build supernetworks where edge lengths correspond to quartet frequencies. We tested the alternative tree topologies within Elateridae and three focal taxa *Plastocerus*, *Drilus*, and *Omalisus* and further, we tested a potential ambiguity of relationships of all these taxa and three putative relatives, i.e., Cantharidae, Lycidae, and Rhagophthalmidae. Resulting supernetworks were visualized in SplitsTree 4.14.6^87^. Further, we used ASTRAL 5.6.1^88^ and genomic dataset to construct coalescent species trees from individual IQ-TREE ML gene topologies at amino acid and nucleotide level.

The focal taxa, *Plastocerus*, *Drilus* and *Omalisus* have been placed in relationships with soft-bodied ‘cantharoid’ families^1–5^. We tested these alternative hypotheses using the densely sampled 66-taxa dataset and compared likelihoods of hypothesized relationships of focal taxa with Cantharidae, Lampyridae and Lycidae and alternatively the topology (*Omalisus*,(*Plastocerus*, *Drilus*)),(Elateridae) which accepts their relationships with Elateridae, but excludes them as sister lineages of Elateridae. The likelihood of these topologies was compared with the best ML topology using AU test^89^ implemented in IQTREE^79^ and using -au option and 1,000,000 replicates.

The click beetle *Melanotus* was earlier recovered as a lineage distantly related to other well-sclerotized Elateridae^8,10^. We encountered a similarly contentious position of *Melanotus*. The topology recovered from the dataset at amino acid level suggested that *Melanotus* does not belong to click beetles^8,10^. Therefore, we estimated the number of genes supporting the alternative relationships. We tested positions of *Melanotus* as (1) a sister to Lampyridae and (2) a sister to other Elateridae by evaluating which single gene partition of amino acid data favor alternative topologies by calculating log-likelihood scores for each gene partition using IQ-TREE option -wpl. As an input we provided both topologies. To interpret the results of the partition log-likelihood and to evaluate the contribution of each gene partition, we calculated differences of each pL score of topologies^90,91^.

## Electronic supplementary material

Supplementary Information

Supplementary Dataset 1

## Data Availability

The DNA sequences reported in this article can be accessed in GenBank under accessions AB123456-789.
