# Supplementary material for: Genome sequences identify three families of Coleoptera as morphologically derived click beetles (Elateridae)
Source: Sci Rep. 2018 Nov 20;8:17084. doi: 10.1038/s41598-018-35328-0 (PMC6244081; doi:10.1038/s41598-018-35328-0)
Supplement: Supplementary file 1 — Supplementary Information [file 41598_2018_35328_MOESM1_ESM.pdf]

Genome sequences identify three families of Coleoptera as morphologically derived click beetles (Elateridae)

D. Kusy, M. Motyka, M. Bocek, A. P. Vogler and L. Bocak

The list of electronic supplementary material

Supplementary table S1. The list of markers in the 95-gene dataset with information on multi-copy genes 2
Supplementary table S2. The list of taxa included in the phylogenomic dataset, accession numbers and the number of sequences available for each taxon 5
Supplementary table S3. The success of ortholog assignment to beetles transcriptomes and proteomes. *Species for which the corresponding 4202 sequences of ortholog groups from available official genes sets were used 6
Supplementary table S4. Overview of official gene sets of six reference species used for transcript ortholog assessment, including the source, version and number of genes. URLs for downloading the data are given. Number of genes correspond with OrthoDB 9.1. 9
Supplementary figure S1. Genomes size estimations for *Drilus, Omalisus* and *Plastocerus* generated by Genomescope, providing a k-mer analysis (k=31) by Jellyfish using filtered data to estimate haploid genome size, heterozygosity and coverage 11
Supplementary figure S2. Heat map of species pairwise amino acid site coverage for partitioned filtered data, supermatrix contains 4202 orthologs generated by AliStat. Shared site coverage is shown from dark blue (low) to white (high) 12
Supplementary figure S3. Heat map of species pairwise amino acid site coverage for partitioned filtered data, supermatrix contains all genes for all taxa generated by AliStat. Shared site coverage is shown from dark blue (low) to white (high) 12
Supplementary figure S4. Heat map of species pairwise nucleotide site coverage for partitioned filtered data, supermatrix contains 4202 orthologs generated by AliStat. Shared site coverage is shown from dark blue (low) to white (high) 13
Supplementary figure S5. Heat map of species pairwise nucleotide site coverage for partitioned filtered data, supermatrix contains all genes for all taxa generated by AliStat. Shared site coverage is shown from dark blue (low) to white (high) 13
Supplementary figure S6. Heat map of species pairwise nucleotide site coverage for the 66-genes dataset generated by AliStat. Shared site coverage is shown from dark blue (low) to white (high) 14
Supplementary figure S7. Heat map of species pairwise nucleotide site coverage for the 66-genes dataset generated by AliStat using only Elateridae taxa. Shared site coverage is shown from dark blue (low) to white (high) 14
Supplementary figure S8. Maximum likelihood (IQ-Tree) topology recovered from 4202 orthologs using partitioned amino acid filtered data 15
Supplementary figure S9. Maximum likelihood (IQ-Tree) topology recovered from 4202 orthologs using partitioned amino acid raw data 16
Supplementary figure S10. Maximum likelihood (IQ-Tree) topology recovered from 2062 orthologs using partitioned filtered amino acid data, supermatrix contains all genes for all taxa 17

Supplementary figure S11. Maximum likelihood (RaxML) topology recovered from 4202 orthologs using unpartitioned amino acid filtered data 18
Supplementary figure S12. Maximum likelihood (IQ-Tree) topology recovered from 4202 orthologs using partitioned nucleotide raw data 19
Supplementary figure S13. Maximum likelihood (IQ-Tree) topology recovered from 2062 orthologs using partitioned filtered nucleotide data, supermatrix contains all genes for all taxa 20
Supplementary figure S14. Maximum likelihood (IQ-Tree) topology recovered from 4202 orthologs using partitioned filtered nucleotide data using only codon positions 1+2 21
Supplementary figure S15. Maximum likelihood (IQ-Tree) topology recovered from 66-genes dataset at nucleotide level using only Elateridae taxa 22
Supplementary figure S16. Maximum likelihood (IQ-Tree) topology recovered from 66-genes dataset at nucleotide level using only Elateridae taxa and Rhagophthalmidae as an outgroup 23
Supplementary figure S17. Maximum likelihood (IQ-Tree) topology recovered from 66-genes dataset at nucleotide level without *Cardiotarsus* (excluded due to high amount of missing data) 24
Supplementary figure S18. Coalescent species tree inferred by ASTRAL from 4202 single genes ML trees inferred by IQ-Tree at nucleotide level 25
Supplementary figure S19. Coalescent species tree inferred by ASTRAL from 4202 single genes ML trees inferred by IQ-Tree at amino acid level 26
Supplementary figure S20. Coalescent species tree inferred by ASTRAL from 66 single genes ML trees inferred by IQ-Tree at nucleotide level 27
Supplementary figure S21. Calculated log-likelihood difference of each single gene partition scores at amino acid level received for (*Melanotus*(Rhagophthalmidae+,Lampyridae)) +(Elateridae) and alternative ((Rhagophthalmidae+Phengodidae)Lampyridae) +(*Melanotus*+Elateridae) topologies 28
Supplementary figure S22. Maximum likelihood IQ-Trees topologies calculated at amino acid level which support (A) (*Melanotus*(Rhagophthalmidae+Lampyridae))+(Elateridae) 1098 genes or alternative (B) ((Rhagophthalmidae+Phengodidae)Lampyridae)+(*Melanotus*+ Elateridae) 959 genes 29
Supplementary figure S23. FcLM analysis of *Omalisus* position based on the amino acid matrix of 4202 filtered and partitioned orthologs 30
Supplementary figure S24. Draft genomes assembly statistics 31

Supplementary Table S1.
The list of markers in the 95-gene dataset with information on multi-copy genes.

Zhang et. al 2018<br>Genes SQ	Otrhology/Paralogy	Occurence in OrthoDB9.1<br>Species (# of gene copies)
Aats-ile	multi-copy	*Onthophagus taurus* (2)
Ace	single-copy	
alphaCOP	single-copy	
alpha-Spec	single-copy	
AP47	single-copy	
Art1	single-copy	
beta'Cop	single-copy	
BOP1	single-copy	
brat	multi-copy	*Dendroctonus ponderosae* (2)
Brel	single-copy	Missing in *Agrilus planipennis*
Bx42	single-copy	
CAD	single-copy	
CadN	single-copy	
calypso	single-copy	
CaP60A	single-copy	
Cdk7	single-copy	
CG11652	single-copy	
CG3999	single-copy	
CG4933	single-copy	
CG6230	multi-copy	*Onthophagus taurus* (2)
CG6512	single-copy	
CG7288	single-copy	
CG7433	multi-copy	*Leptinotarsa decemlineata* (4), *Agrilus planipennis* (3), *Anoplophora glabripennis* (3) *Onthophagus taurus* (3), *Dendroctonus ponderosae* (3), *Tribolium castaneum* (3)
CG7470	multi-copy	*Agrilus planipennis*, *Anoplophora glabripennis*, *Leptinotarsa decemlineata*
CG8545	single-copy	
CG9518	multi-copy	*Agrilus planipennis* (25), *Anoplophora glabripennis* (14), *Leptinotarsa decemlineata* (20), *Onthophagus taurus* (10), *Dendroctonus ponderosae* (16), *Tribolium castaneum* (21)
ck	multi-copy	*Agrilus planipennis* (2), *Onthophagus taurus* (2), *Leptinotarsa decemlineata* missing
Crc	single-copy	
dbo	single-copy	
Dhc98D	single-copy	
DIP2	multi-copy	*Agrilus planipennis* (2), *Onthophagus taurus* (2), *Leptinotarsa decemlineata* (2)
dnc	single-copy	
DopR2	single-copy	

dyl	single-copy	
Elp3	single-copy	
Emb	single-copy	
FBXO11	single-copy	
fz2	single-copy	
Gapdh2	single-copy	
Glus	multi-copy	*Anoplophora glabripennis* (2), *Leptinotarsa decemlineata* (2)
Hem	single-copy	
Hmgs	single-copy	
Hr38	single-copy	
Hsc70-5	single-copy	
hts	multi-copy	*Dendroctonus ponderosae* (2)
ico	single-copy	
Idh	single-copy	
Int6	single-copy	
IntS11	single-copy	
Inx2	multi-copy	*Dendroctonus ponderosae* (2)
Ip259	multi-copy	*Dendroctonus ponderosae* (3)
l(2)37Cb	multi-copy	*Dendroctonus ponderosae* (2), *Leptinotarsa decemlineata* (2)
l(3)72Ab	multi-copy	*Agrilus planipennis* (2), *Anoplophora glabripennis* (2), *Leptinotarsa decemlineata* (2), *Onthophagus taurus*(2), *Dendroctonus ponderosae* (3), *Tribolium castaneum* (2)
Lar	single-copy	
Lon	multi-copy	*Agrilus planipennis* (2), *Leptinotarsa decemlineata* (2)
mor	multi-copy	*Dendroctonus ponderosae* (2)
Mtpalpha	multi-copy	*Dendroctonus ponderosae* (2)
Ndae1	single-copy	
nej	multi-copy	*Dendroctonus ponderosae* (2)
nero	single-copy	
Nhel	single-copy	missing *Tribolium castaneum*
nito	single-copy	
nonC	single-copy	
Notch	single-copy	
Nrx-IV	single-copy	
Past1	single-copy	
PlexA	multi-copy	*Anoplophora glabripennis* (2)
Rbcn-3A	multi-copy	*Leptinotarsa decemlineata* (5)
RnrL	single-copy	
rols	multi-copy	*Leptinotarsa decemlineata* (3)
RpII140	single-copy	
RpII215	multi-copy	*Anoplophora glabripennis* (2)
Rpn1	single-copy	
Rpn3	single-copy	
Rpn6	multi-copy	*Agrilus planipennis* (2), *Anoplophora glabripennis* (2), *Leptinotarsa*

		decemlineata (3), *Onthophagus taurus* (2), *Tribolium castaneum* (2)
Sec24	multi-copy	*Dendroctonus ponderosae* (2)
sec71	single-copy	
Shal	single-copy	
Shot	single-copy	
SIPA1L1	single-copy	
Spt6	single-copy	
Su(H)	single-copy	
Sur-8	single-copy	
sxc	single-copy	
Taf2	single-copy	
TfIIEalpha	single-copy	
Tmp	multi-copy	*Agrilus planipennis* (5), *Anoplophora glabripennis* (2), *Leptinotarsa decemlineata* (5), *Onthophagus taurus* (3), *Dendroctonus ponderosae* (6), *Tribolium castaneum* (3)
Top2	single-copy	
Tor	multi-copy	*Onthophagus taurus* (3), *Leptinotarsa decemlineata* (3), *Agrilus planipennis* (2)
Trpml	single-copy	
Vacht	single-copy	
VGAT	single-copy	
WDR44	multi-copy	*Onthophagus taurus* (2)
wls	single-copy	
zip	single-copy	

Supplementary Table S2. The list of taxa included in the phylogenomic dataset, accession and the number of sequences available for each taxon.

Species	Accession	# of seq.	Download	Date	Reference
Outgroups:					
Onthophagus taurus (ref)	PRJNA167478	17483	i5K	5.3.2017	1
Tribolium castaneum (ref)	PRJNA12540	16631	iBeetle	5.3.2017	2,3
Dendroct. ponderosae (ref)	PRJNA360270	13088	ENS Metazoa	5.3.2017	4
Anoploph. glabripennis (ref)	PRJNA167479	22035	i5K	5.3.2017	5
Leptinotarsa decemlin. (ref)	PRJNA171749	24671	i5K	5.3.2017	1
Buprestoidea					
Agrilus planipennis (ref)	PRJNA230921	15497	i5K	5.3.2017	1
Elateroidea					
Chauliognathus flavipes	PRJNA347807	92143	NCBI,SRA	5.3.2017	6
Rhagophthalmus sp.	PRJNA339505	38989	NCBI,SRA	5.3.2017	7
Phrixothrix hirtus	RJNA347807	31428	NCBI,SRA	5.3.2017	6
Photinus pyralis	PRJNA321737	174087	NCBI,SRA	5.3.2017	8
Assymetricata circumdata	PRJNA339505	55590	NCBI,SRA	10.1.2017	7
Aquatica ficta	PRJNA339505	70558	NCBI,SRA	10.1.2017	7
Pyrocoelia pectoralis	PRJNA339505	76908	NCBI,SRA	10.1.2017	7
Melanotus cribricollis	PRJNA417752	38705	NCBI,SRA	15.4.2017	9
Ignelater luminosus	PRJNA418169	27553	fireflybase.org	15.4.2017	10
Platerodrilus sp.	ABC1234567	33998		this study	
Drilus mauritanicus	ABC1234567	98819		this study	
Omalisus fontisbellaquei	ABC1234567	39344		this study	
Plastocerus angulosus	ABC1234567	53820		this study	

(ref) - reference taxon

References

1 Poelchau M et al. 2014 The i5k Workspace@NAL‚Äîenabling genomic data access, visualization and curation of arthropod genomes. Nucleic Acids Research, 43(D1). doi:10.1093/nar/gku983

2 Shelton JM et al. 2015 Tools and pipelines for BioNano data: molecule assembly pipeline and FASTA super scaffolding tool. BMC Genomics, 16(1). doi:10.1186/s12864-015-1911-8

3 Richards S et al. 2008 The genome of the model beetle and pest *Tribolium castaneum*. Nature, 452(7190), 949-955. doi:10.1038/nature06784

4 Keeling CI et al. 2013 Draft genome of the mountain pine beetle, *Dendroctonus ponderosae* Hopkins, a major forest pest. Genome Biology, 14(3). doi:10.1186/gb-2013-14-3-r27

5 McKenna DD et al. 2017 Genome of the Asian longhorned beetle (*Anoplophora glabripennis*), a globally significant invasive species, reveals key functional and evolutionary innovations at the beetle‚Äìplant interface. Genome Biology 2017 17(1), 227. DOI: 10.1186/s13059-016-1088-8

6 Amaral, DT et al. 2017 Transcriptional comparison of the photogenic and non-photogenic tissues of *Phrixothrix hirtus* (Coleoptera: Phengodidae) and non-luminescent

Chauliognathus flavipes (Coleoptera: Cantharidae) give insights on the origin of lanterns in railroad worms. Gene Reports, 7, 78-86. doi:10.1016/j.genrep.2017.02.004
7 Wang K et al. 2017 Transcriptome sequencing and phylogenetic analysis of four species of luminescent beetles. Scientific Reports, 7(1). doi:10.1038/s41598-017-01835-9
8 Fallon et al. 2016 Sulfoluciferin is Biosynthesized by a Specialized Luciferin Sulfotransferase in Fireflies. Biochemistry, 55(24), 3341-3344. doi:10.1021/acs.biochem.6b00402
9 Ye, B, Zhang Y, Shu J, Wu H, Wang H. 2018 RNA-sequencing analysis of fungi-induced transcripts from the bamboo wireworm *Melanotus cribricollis* (Coleoptera: Elateridae) larvae. PLoS One 13, e019118. (doi:10.1371/journal.pone.0191187)
10 Fallon TR,et al. 2017 Firefly genomes illuminate parallel origins of bioluminescence in beetles. URL: https://www.biorxiv.org/content/biorxiv/early/2018/02/25/237586.full.pdf. Accessed on June 22 nd , 2018. (doi:10.1101/237586)

Supplementary Table S3. The success of ortholog assignment to beetles transcriptomes and proteomes. *Species for which the corresponding 4202 sequences of ortholog groups from available official genes sets were used.

Species name	Assigned ortholog groups	Successfully assigned ortholog groups after removal of outliers
Drilus	3978	3964
Melanotus cribricollis	3683	3674
Plastocerus	3960	3941
Omalisus	3918	3894
Pyrocoelia	3767	3758
Platerodrilus sp.	3283	3263
Asymmetricata	3603	3589
Aquatica	3703	3693
Phrixothrix hirtus	2878	2855
Chauliognathus flavipes	3669	3619
Rhagophthalmus sp.	3769	3762
Photinus pyralis	3403	3385
Ignelater luminosus	3869	3861
*Agrilus planipennis**	4202	4202
*Anoplophora glabripennis**	4202	4202
*Dendroctonus ponderosae**	4202	4202
*Leptinotarsa decemlineata**	4202	4202
*Onthophagus taurus**	4202	4202
*Tribolium castaneum**	4202	4202

Supplementary Table S4. Overview of official gene sets of six reference species used for transcript ortholog assessment, including the source, version and number of genes. URLs for downloading the data are given below. Number of genes correspond with OrthoDB 9.1.

Species	Acronym	Source	Version	Download date	Number of genes	Reference
Agrilus planipennis	APLA	i5K	0.5.3	5.3.2017	15 497	1
Anopl. glabripennis	AGLA	i5K	0.5.3	5.3.2017	22 035	2
Dendr. ponderosae	YQE	Ensembl Metazoa	1.0	5.3.2017	13 088	3
Lept. decemlineata	LDEC	i5K	0.5.3	5.3.2017	24 671	1
Ontophagus taurus	OTAU	i5K	0.5.3	5.3.2017	17 483	1
Tribol. castaneum	TC	iBeetle-Base	5.2	5.3.2017	16 631	4,5

References

1 Poelchau, M. et al. (2014). The i5k Workspace@NAL—enabling genomic data access, visualization and curation of arthropod genomes. Nucleic Acids Research, 43(D1). doi:10.1093/nar/gku983

2 McKenna, D. D. et al. Genome of the Asian longhorned beetle (Anoplophora glabripennis), a globally significant invasive species, reveals key functional and evolutionary innovations at the beetle–plant interface. Genome Biology 2017 17(1), 227. DOI: 10.1186/s13059-016-1088-8

3 Keeling, C. I. et al. (2013). Draft genome of the mountain pine beetle, Dendroctonus ponderosae Hopkins, a major forest pest. Genome Biology, 14(3). doi:10.1186/gb-2013-14-3-r27

4 Shelton, J. M. et al. (2015). Tools and pipelines for BioNano data: molecule assembly pipeline and FASTA super scaffolding tool. BMC Genomics, 16(1). doi:10.1186/s12864-015-1911-8

5 Richards, S. et al. (2008). The genome of the model beetle and pest Tribolium castaneum. Nature, 452(7190), 949-955. doi:10.1038/nature06784

Tribolium castaneum

http://bioinf.uni-greifswald.de/tcas/genes/tcas5_annotation/Tcas5.2_GenBank.corrected_v5.renamed.aa

http://bioinf.uni-greifswald.de/tcas/genes/tcas5_annotation/Tcas5.2_GenBank.corrected_v5.renamed.codingseq

Onthophagus taurus

https://i5k.nal.usda.gov/data/Arthropoda/onttau-%28Onthophagus_taurus%29/Current%20Genome%20Assembly/2.Official%20or%20Primary%20Gene%20Set/BCM_version_0.5.3/consensus_gene_set/OTAU.faa

https://i5k.nal.usda.gov/data/Arthropoda/onttau-%28Onthophagus_taurus%29/Current%20Genome%20Assembly/2.Official%20or%20Primary%20Gene%20Set/BCM_version_0.5.3/consensus_gene_set/OTAU.CDS.fna

Anoplophora glabripennis
https://i5k.nal.usda.gov/data/Arthropoda/anogla-%28Anoplophora_glabripennis%29/Current%20Genome%20Assembly/3.Additional%20Gene%20Sets%20and%20Annotation%20Projects/BCM_version_0.5.3-Primary_Gene_Set/primary_gene_set/AGLA.CDS.fna.gz
https://i5k.nal.usda.gov/data/Arthropoda/anogla-%28Anoplophora_glabripennis%29/Current%20Genome%20Assembly/3.Additional%20Gene%20Sets%20and%20Annotation%20Projects/BCM_version_0.5.3-Primary_Gene_Set/primary_gene_set/AGLA.faa.gz
Agrilus planipennis
https://i5k.nal.usda.gov/data/Arthropoda/agrpla-%28Agrilus_planipennis%29/Current%20Genome%20Assembly/2.Official%20or%20Primary%20Gene%20Set/BCM_version_0.5.3/consensus_gene_set/APLA.faa
https://i5k.nal.usda.gov/data/Arthropoda/agrpla-%28Agrilus_planipennis%29/Current%20Genome%20Assembly/2.Official%20or%20Primary%20Gene%20Set/BCM_version_0.5.3/consensus_gene_set/APLA.CDS.fna
Dendroctonus ponderosae
ftp://ftp.ensemblgenomes.org/pub/metazoa/release-37/fasta/dendroctonus_ponderosae/cds/Dendroctonus_ponderosae.DendPond_male_1.0.cds.all.fa.gz
ftp://ftp.ensemblgenomes.org/pub/metazoa/release-37/fasta/dendroctonus_ponderosae/pep/Dendroctonus_ponderosae.DendPond_male_1.0.pep.all.fa.gz
Leptinotarsa decemlineata
https://i5k.nal.usda.gov/data/Arthropoda/lepdec-%28Leptinotarsa_decemlineata%29/Current%20Genome%20Assembly/2.Official%20or%20Primary%20Gene%20Set/BCM_version_0.5.3-Primary_Gene_Set/primary_gene_set/LDEC.faa.gz
https://i5k.nal.usda.gov/data/Arthropoda/lepdec-%28Leptinotarsa_decemlineata%29/Current%20Genome%20Assembly/2.Official%20or%20Primary%20Gene%20Set/BCM_version_0.5.3-Primary_Gene_Set/primary_gene_set/LDEC.CDS.fna.gz

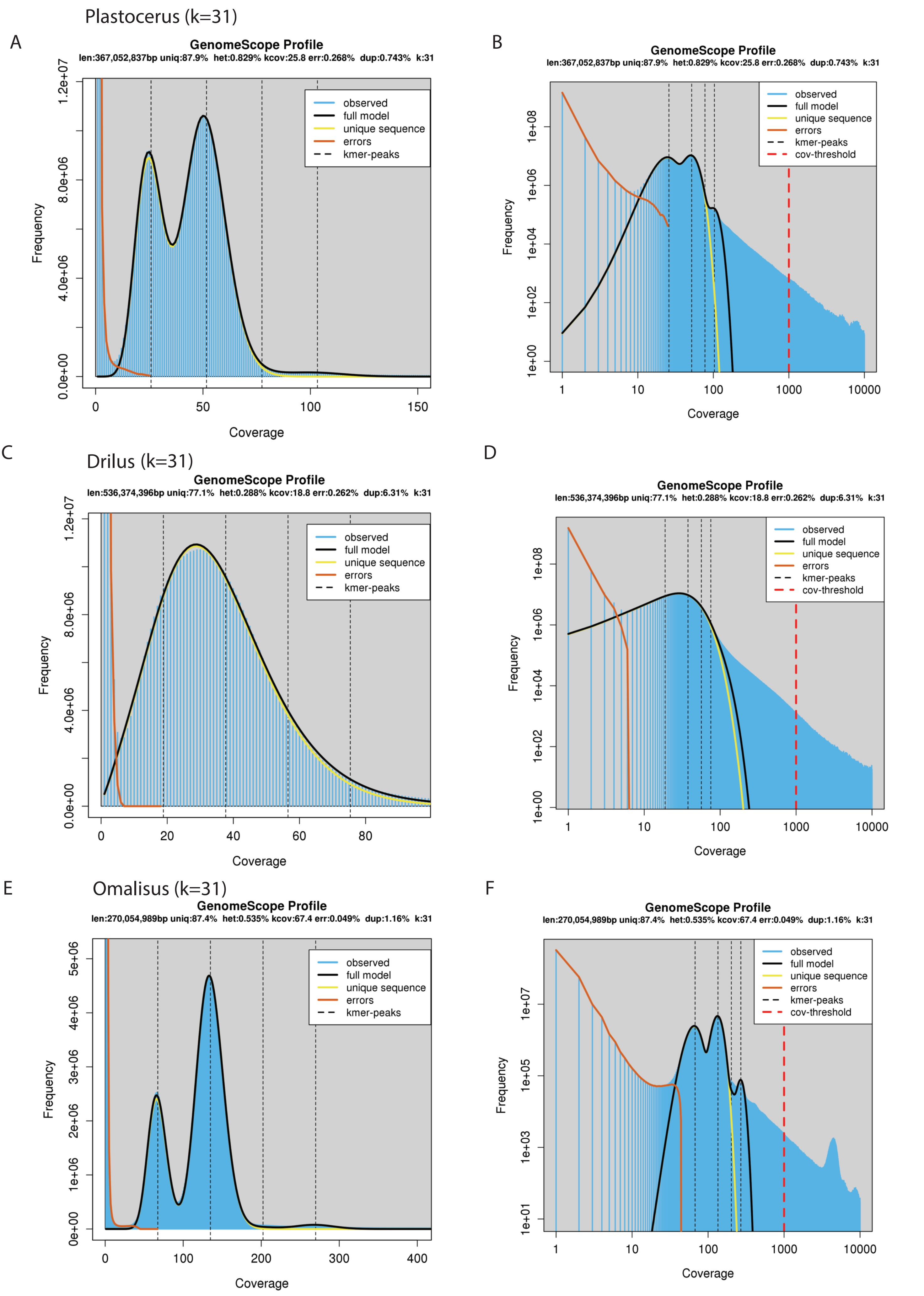

Supplementary figure S1. Genomes size estimations for Drilus, Omalisus and Plastocerus generated by S Genomescope, providing a k-mer analysis (k=31) by Jellyfish using filtered data to estimate haploid genome size, heterozygosity and coverage.

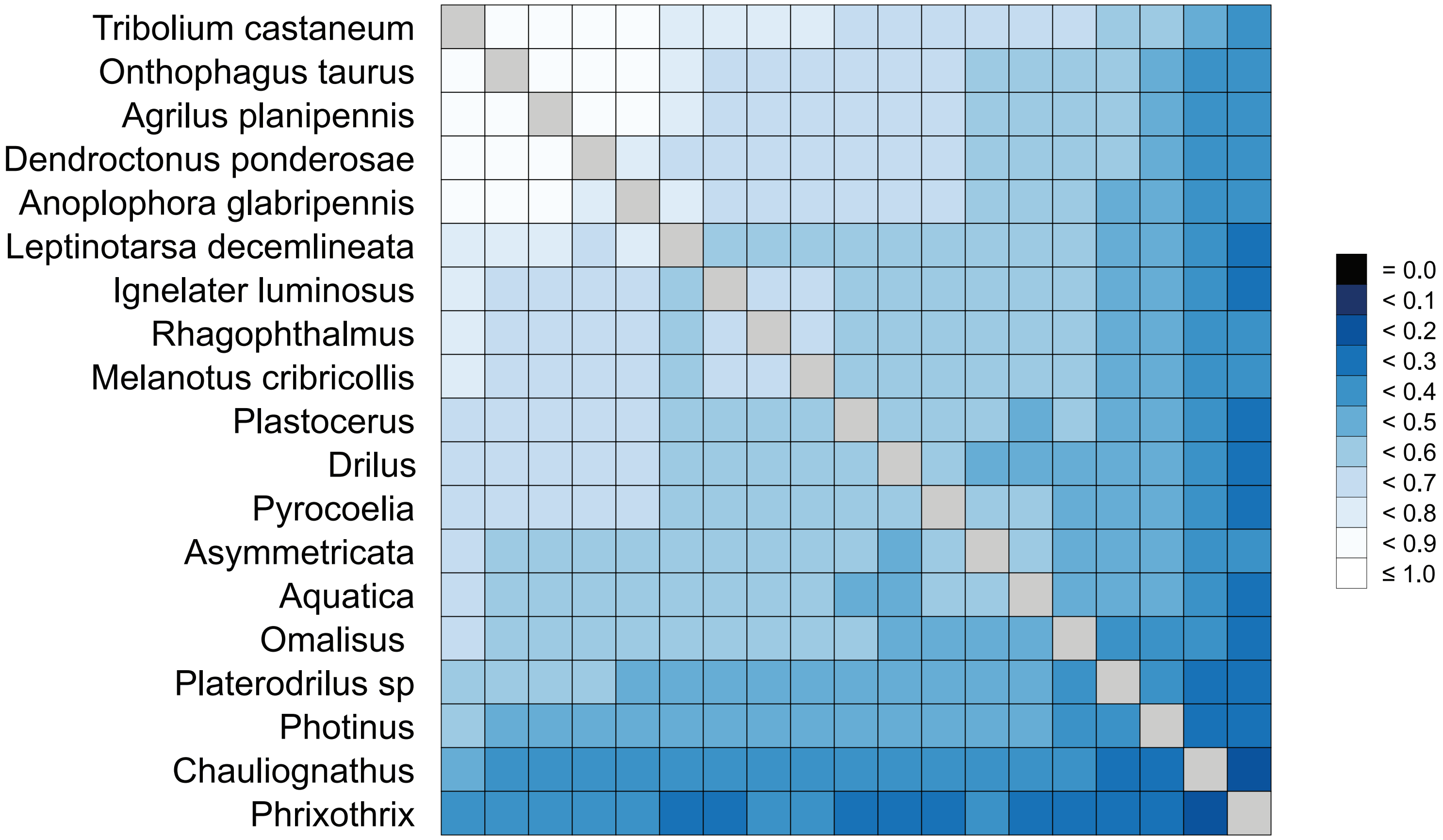

Supplementary figure S2. Heat map of species pairwise amino acid site coverage for partitioned filtered data, supermatrix contains 4202 orthologs generated by AliStat. Shared site coverage is shown from dark blue (low) to white (high).

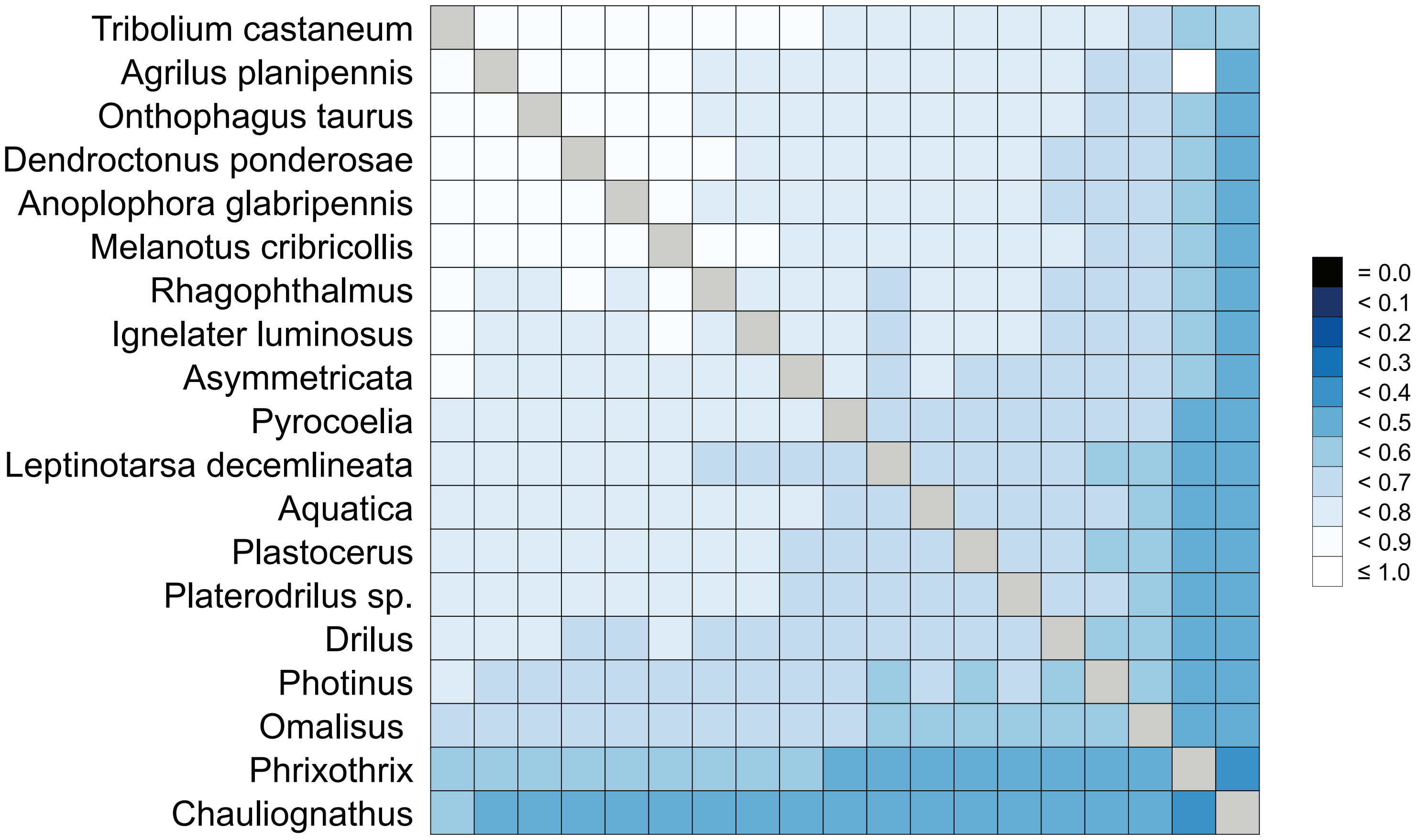

Supplementary figure S3. Heat map of species pairwise amino acid site coverage for partitioned filtered data, supermatrix contains all genes for all taxa generated by AliStat. Shared site coverage is shown from dark blue (low) to white (high).

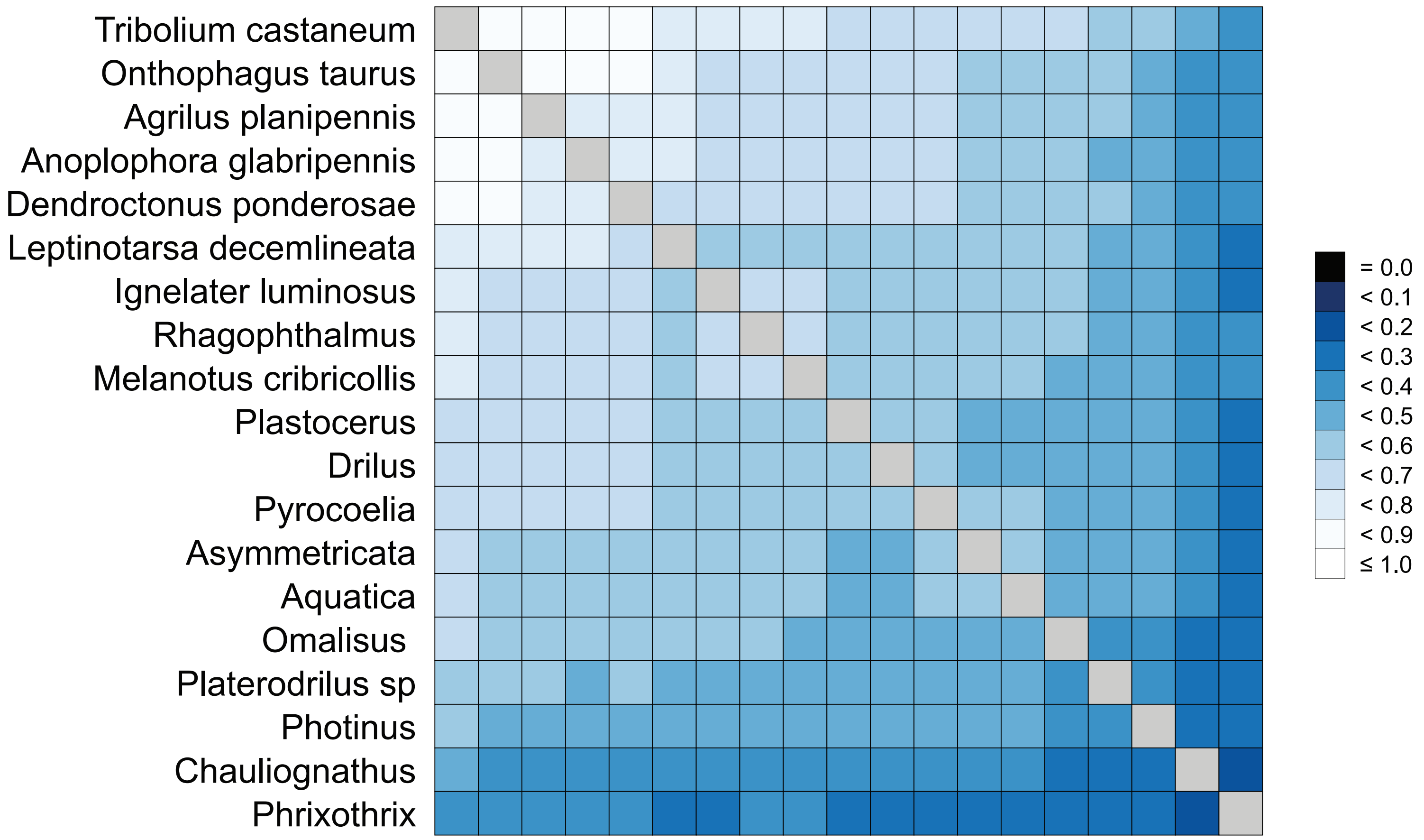

Supplementary figure S4. Heat map of species pairwise nucleotide site coverage for partitioned filtered data, supermatrix contains 4202 orthologs generated by AliStat. Shared site coverage is shown from dark blue (low) to white (high).

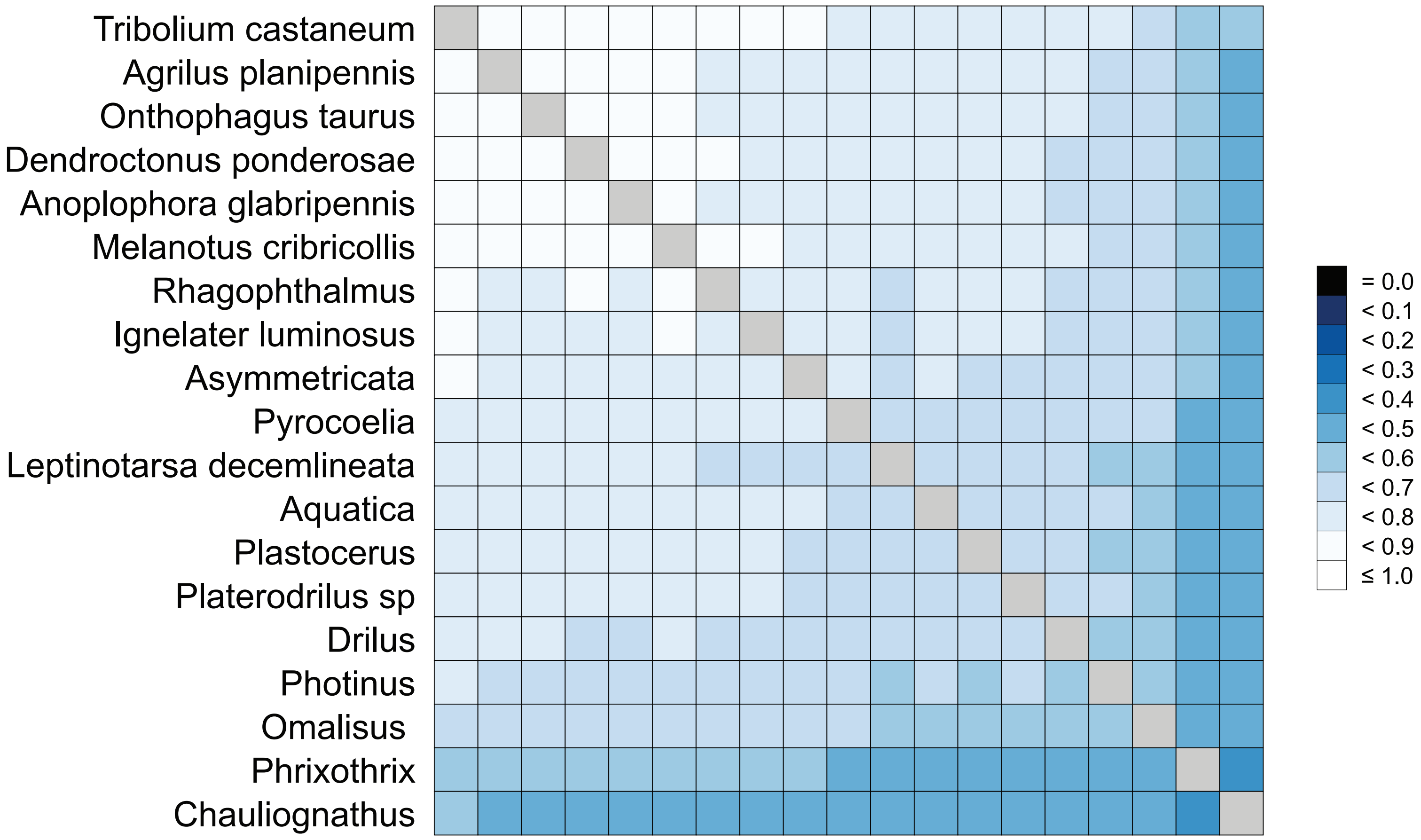

Supplementary figure S5. Heat map of species pairwise nucleotide site coverage for partitioned filtered data, supermatrix contains all genes for all taxa generated by AliStat. Shared site coverage is shown from dark blue (low) to white (high).

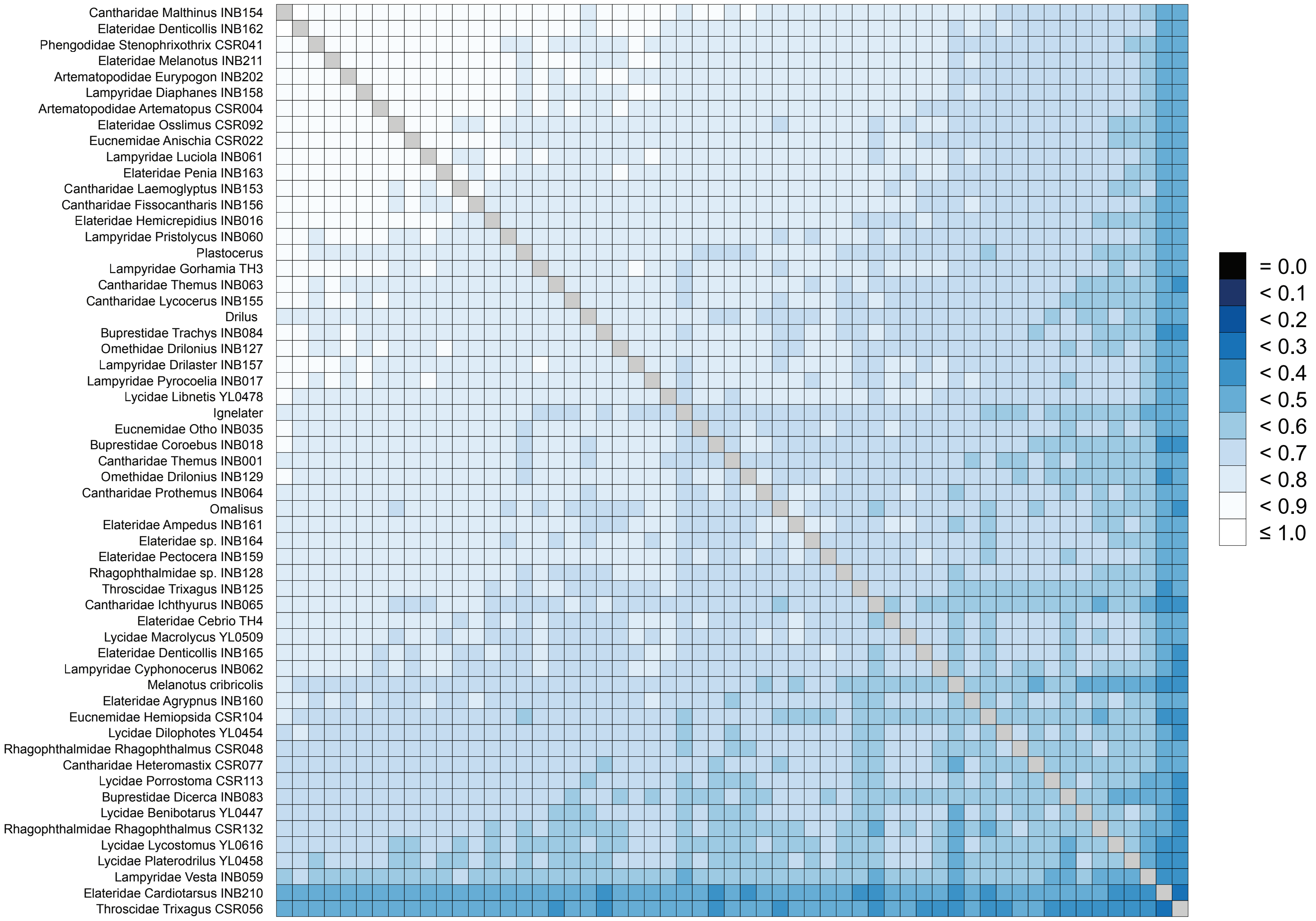

Supplementary figure S6. Heat map of species pairwise nucleotide site coverage for the 66-genes dataset generated by AliStat. Shared site coverage is shown from dark blue (low) to white (high).

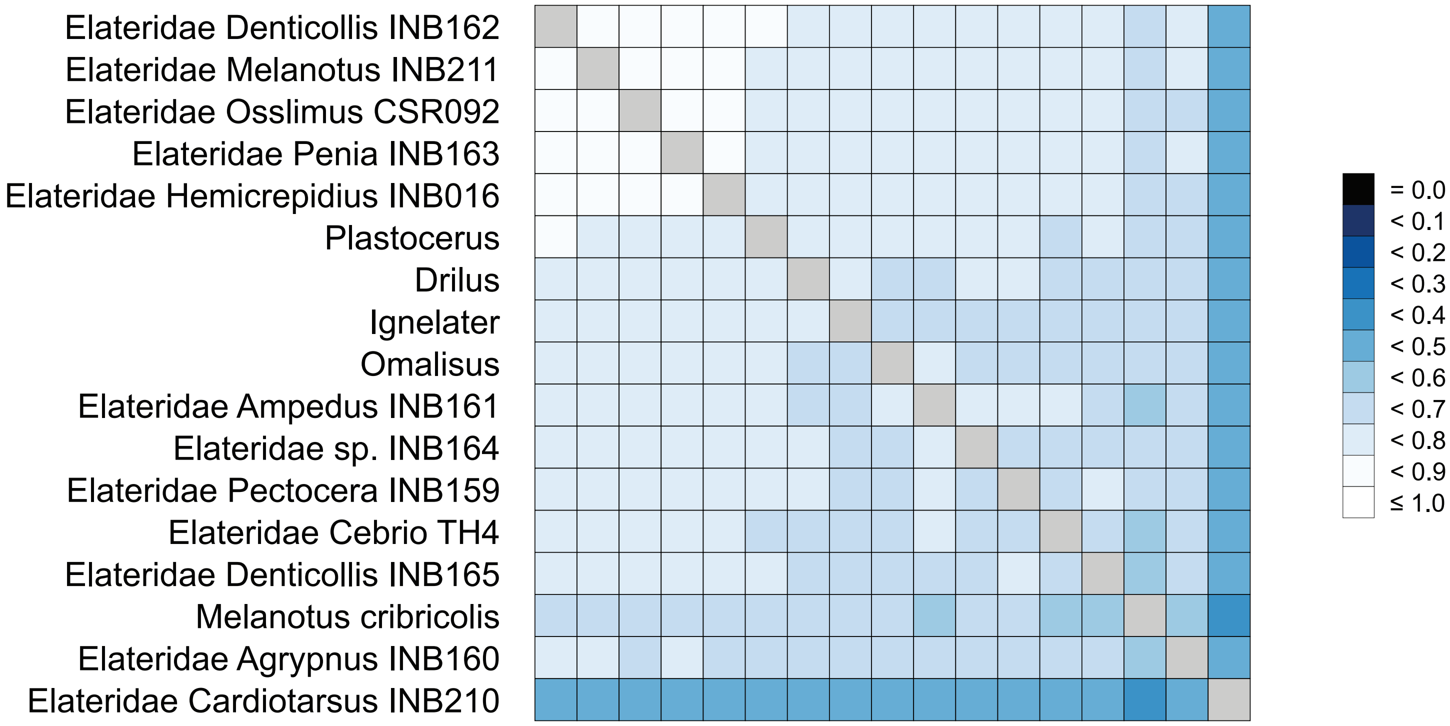

Supplementary figure S7. Heat map of species pairwise nucleotide site coverage for the 66-genes dataset generated by AliStat using only Elateridae taxa. Shared site coverage is shown from dark blue (low) to white (high).

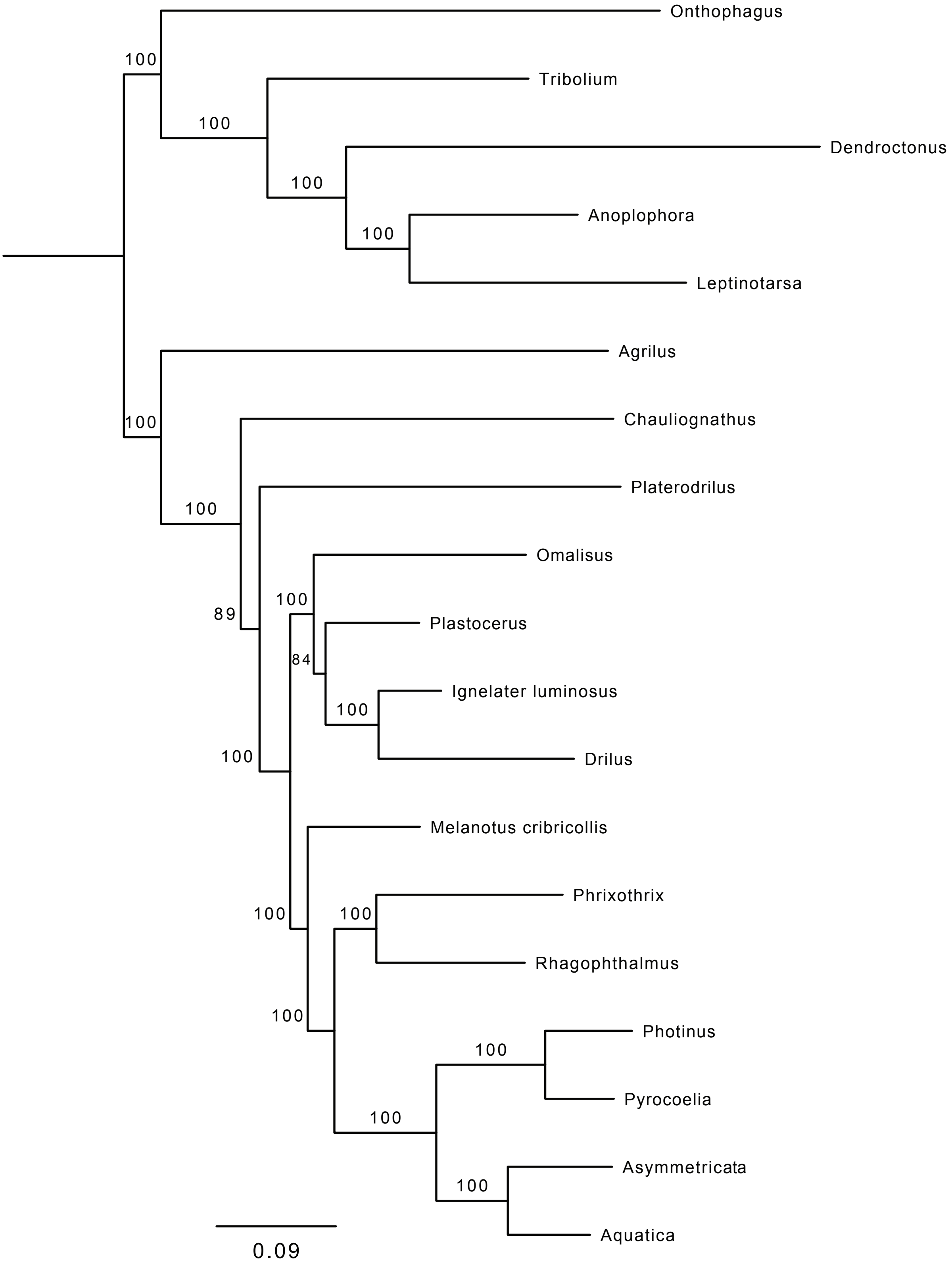

Supplementary figure S8. Maximum likelihood (IQ-Tree) topology recovered from 4202 orthologs using partitioned amino acid filtered data.

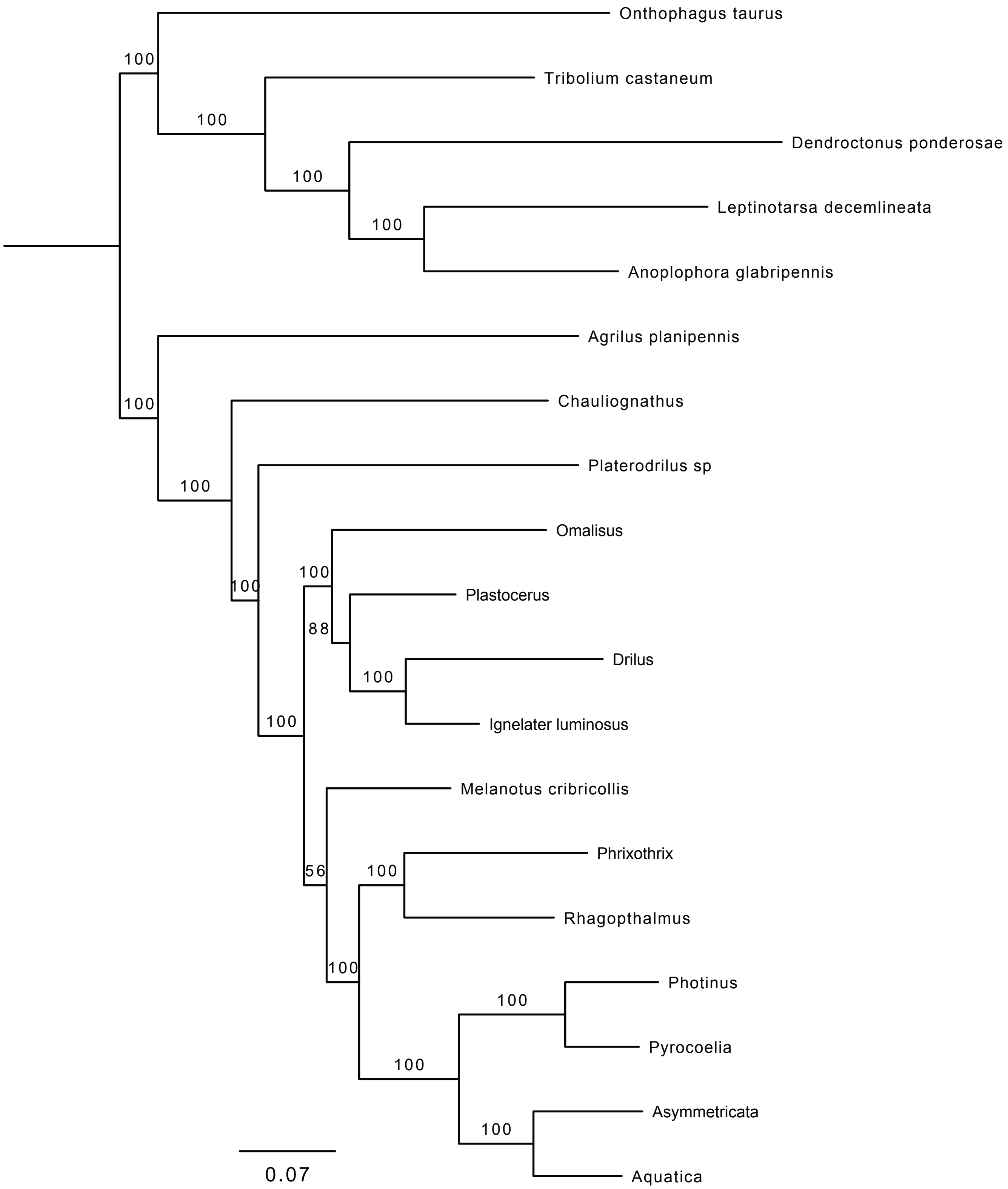

Supplementary figure S9. Maximum likelihood (IQ-Tree) topology recovered from 4202 orthologs using partitioned amino acid raw data.

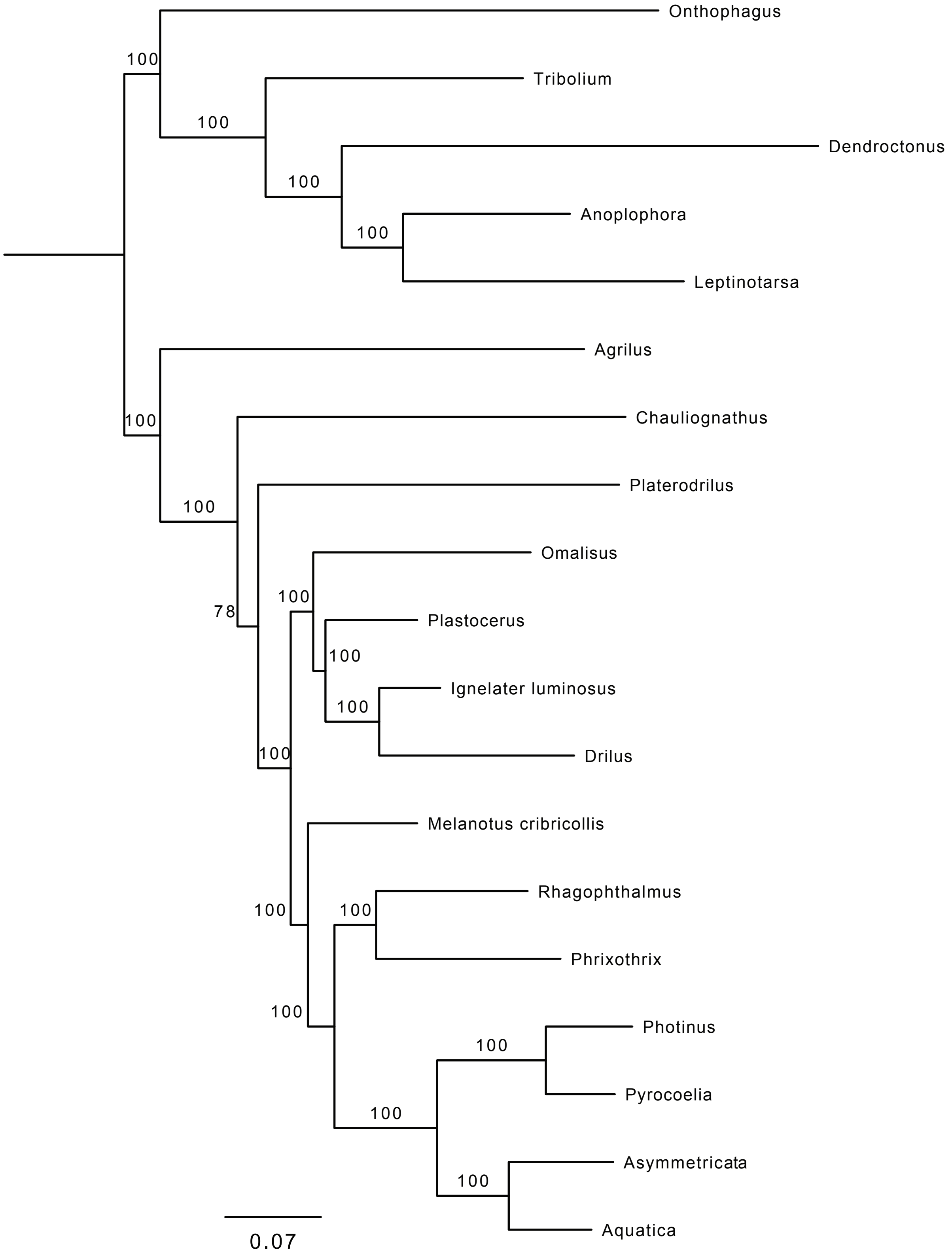

Supplementary figure S10. Maximum likelihood (IQ-Tree) topology recovered from 2062 orthologs using partitioned filtered amino acid data, supermatrix contains all genes for all taxa.

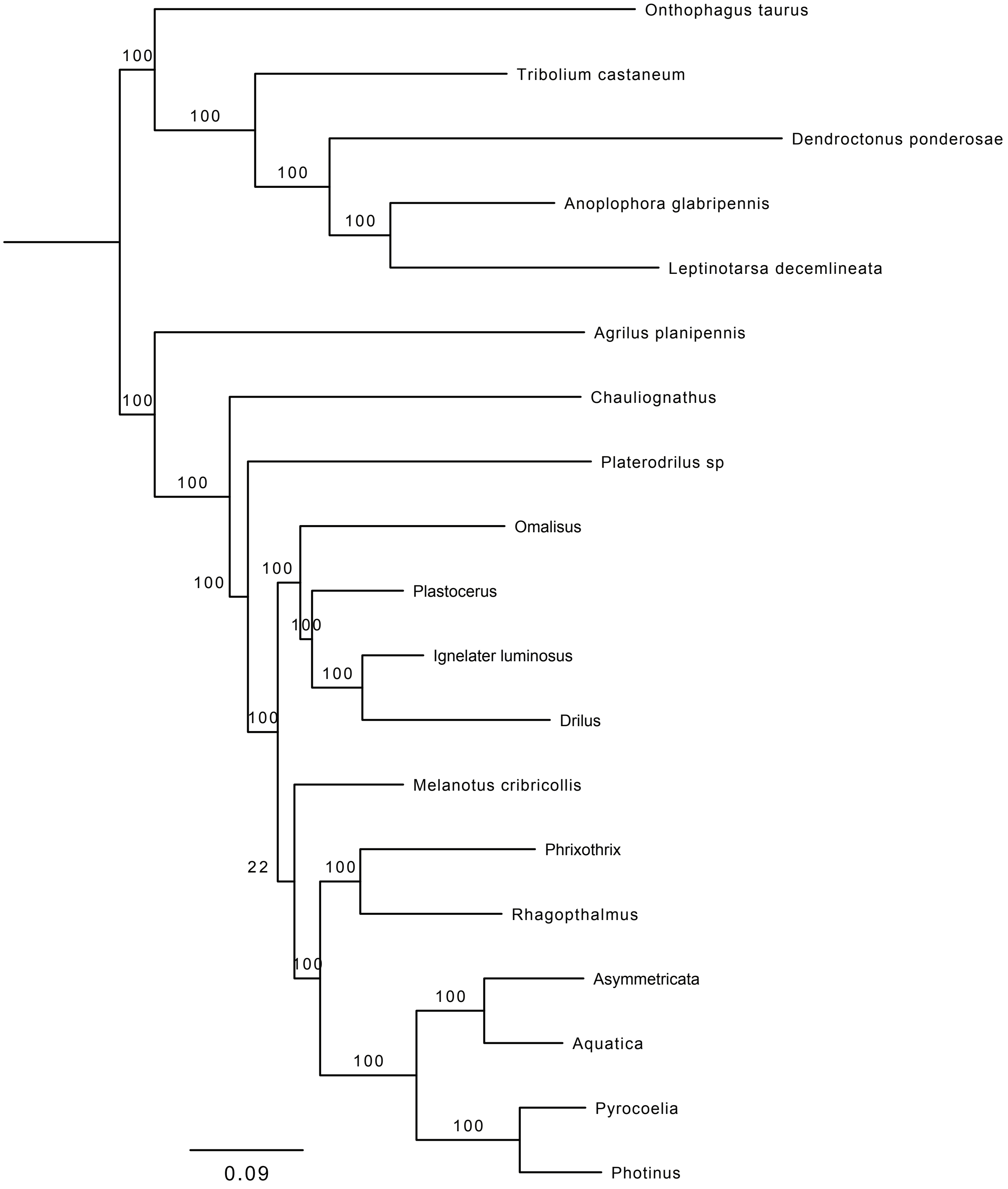

Supplementary figure S11. Maximum likelihood (RaxML) topology recovered from 4202 orthologs using unpartitioned amino acid filtered data.

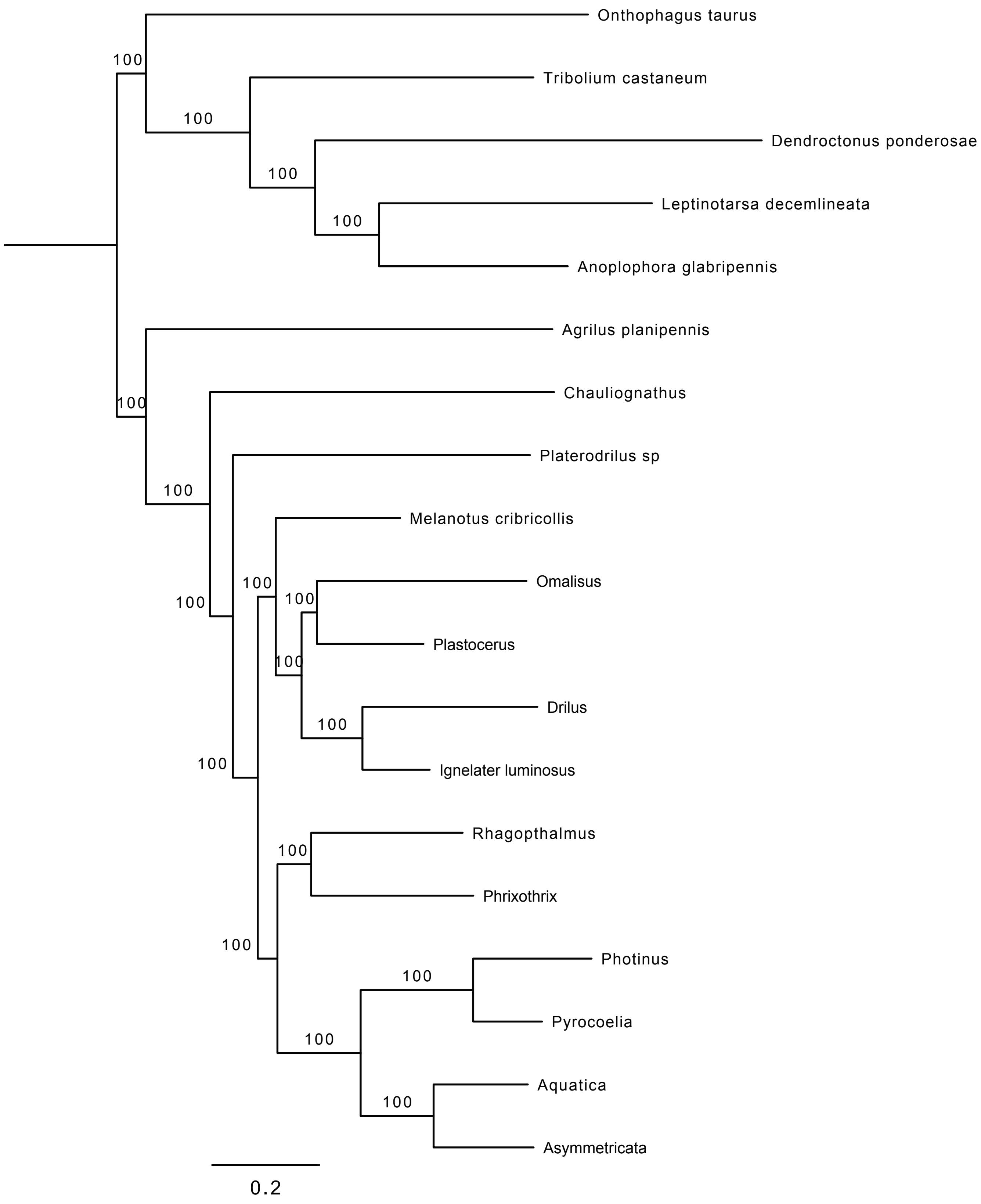

Supplementary figure S12. Maximum likelihood (IQ-Tree) topology recovered from 4202 orthologs using partitioned nucleotide raw data.

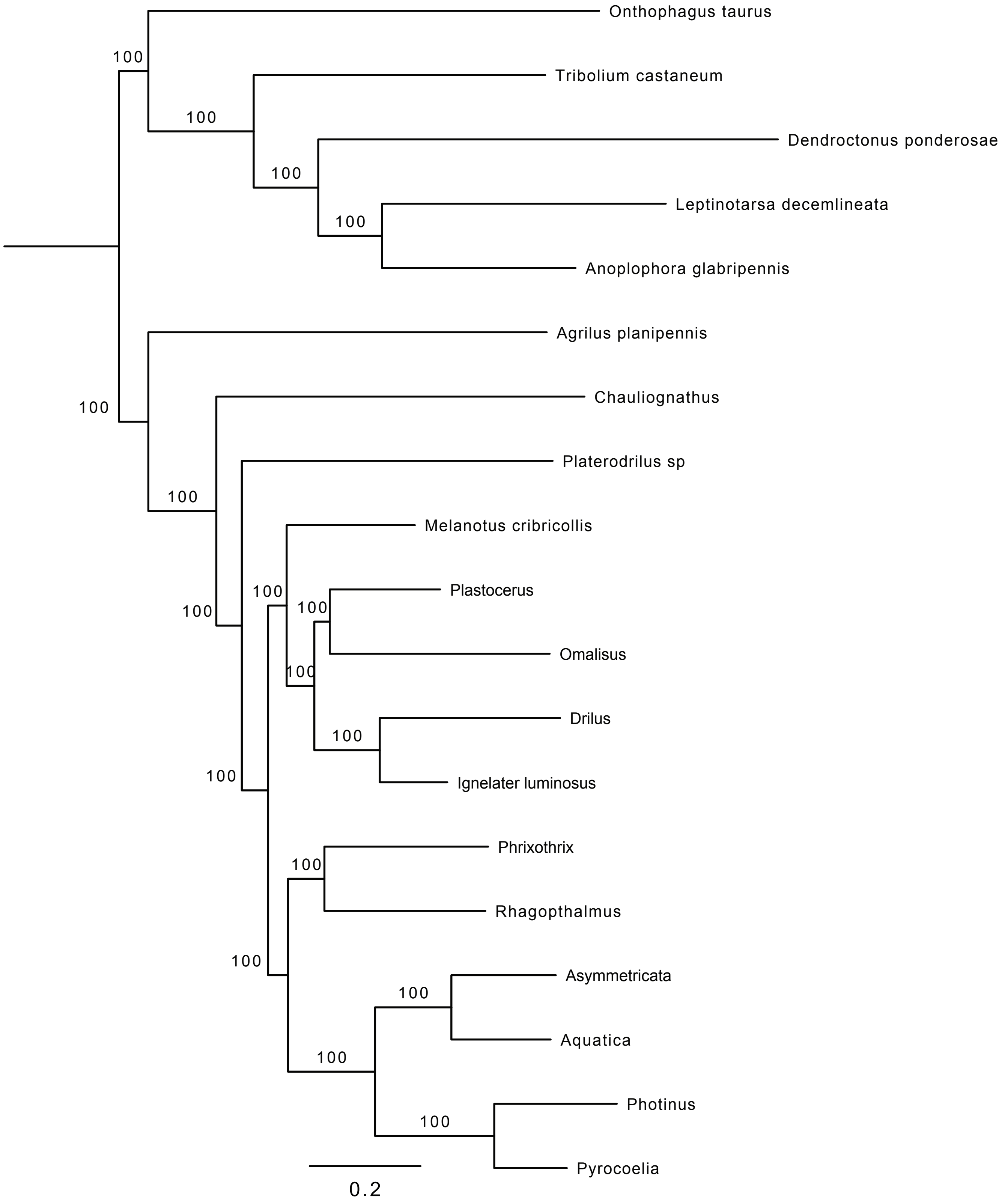

Supplementary figure S13. Maximum likelihood (IQ-Tree) topology recovered from 2062 orthologs using partitioned filtered nucleotide data, supermatrix contains all genes for all taxa.

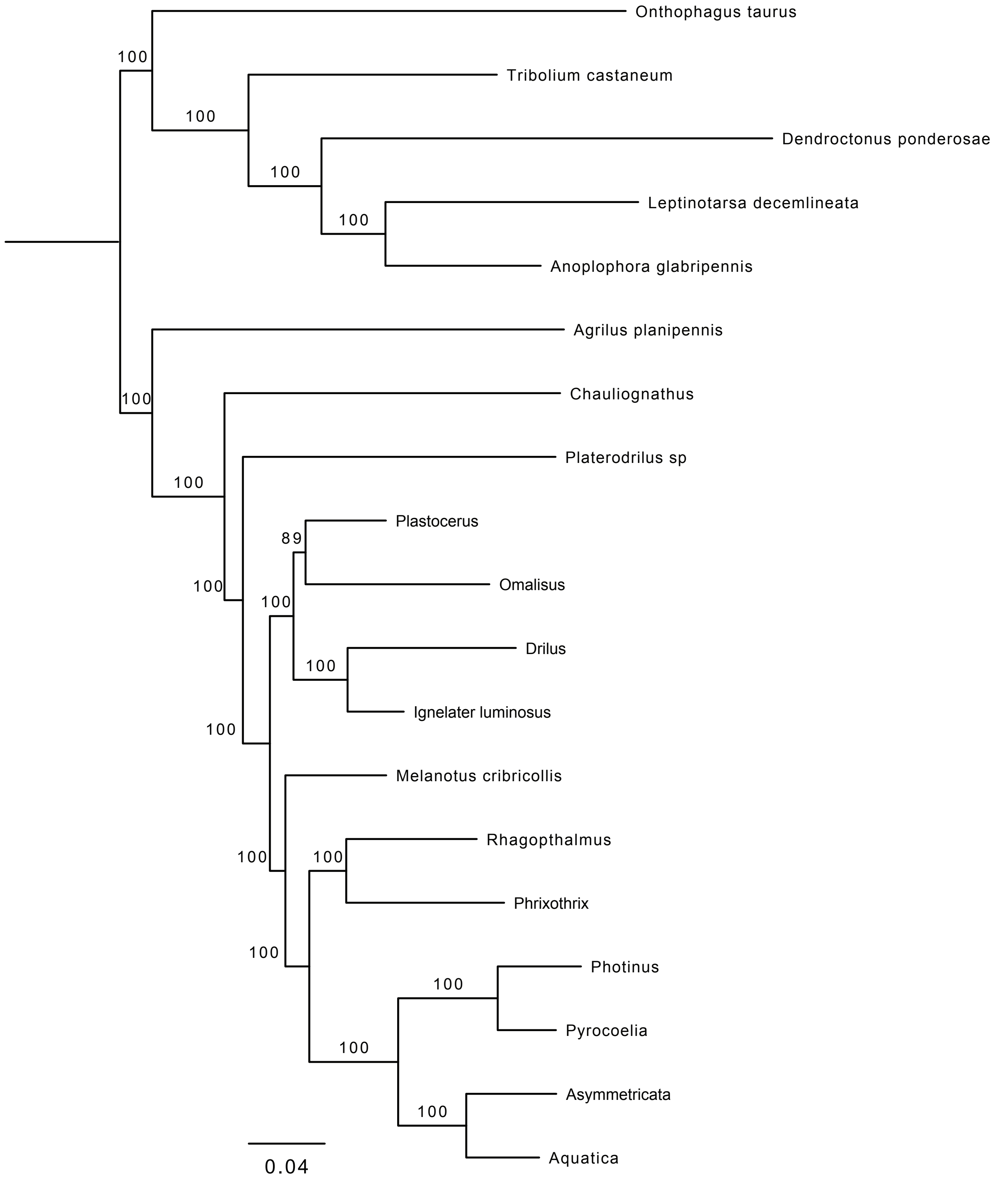

Supplementary figure S14. Maximum likelihood (IQ-Tree) topology recovered from 4202 orthologs using partitioned filtered nucleotide data using only codon positions 1+2.

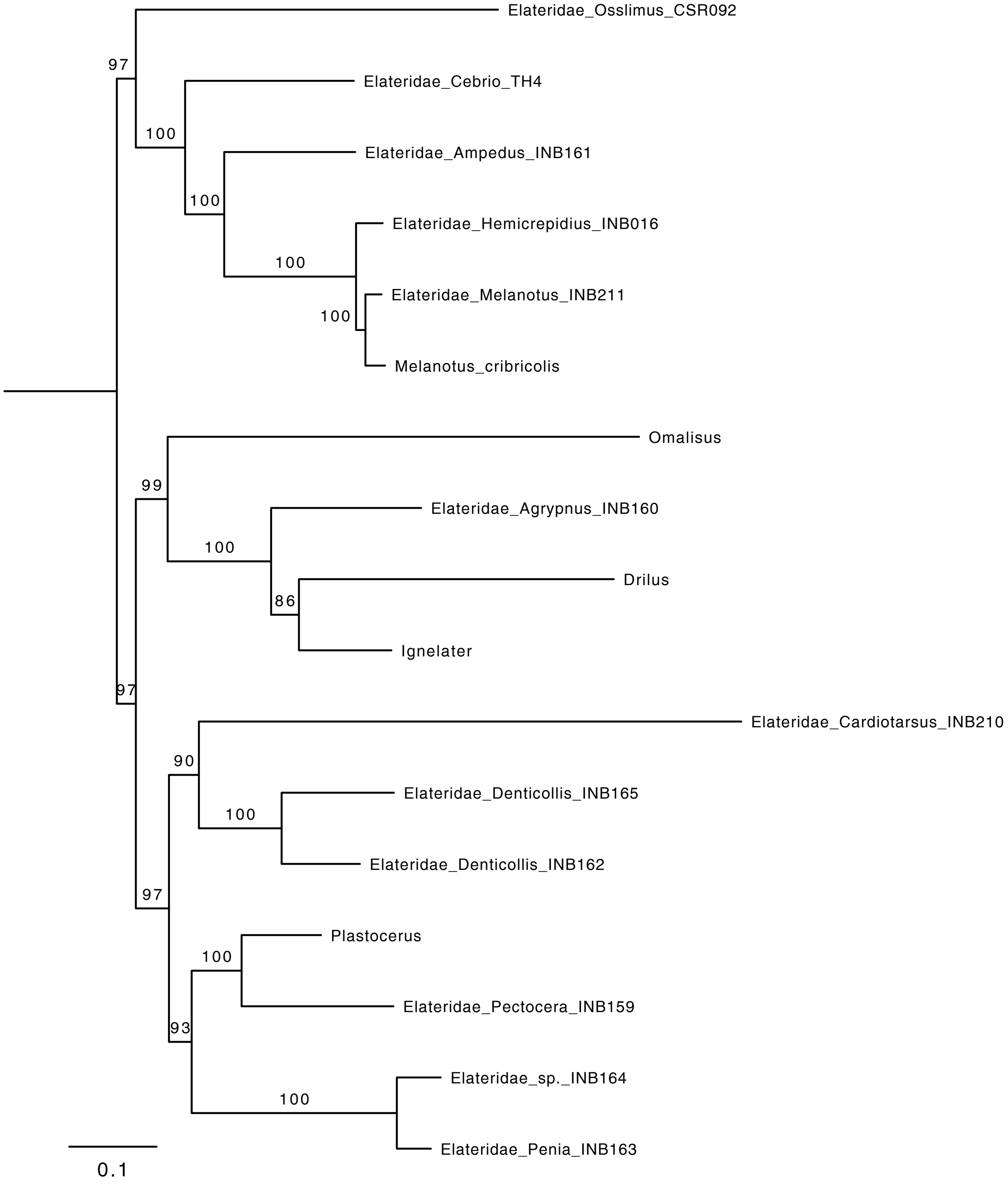

Supplementary figure S15. Maximum likelihood (IQ-Tree) topology recovered from 66-genes dataset at nucleotide level using only Elateridae taxa.

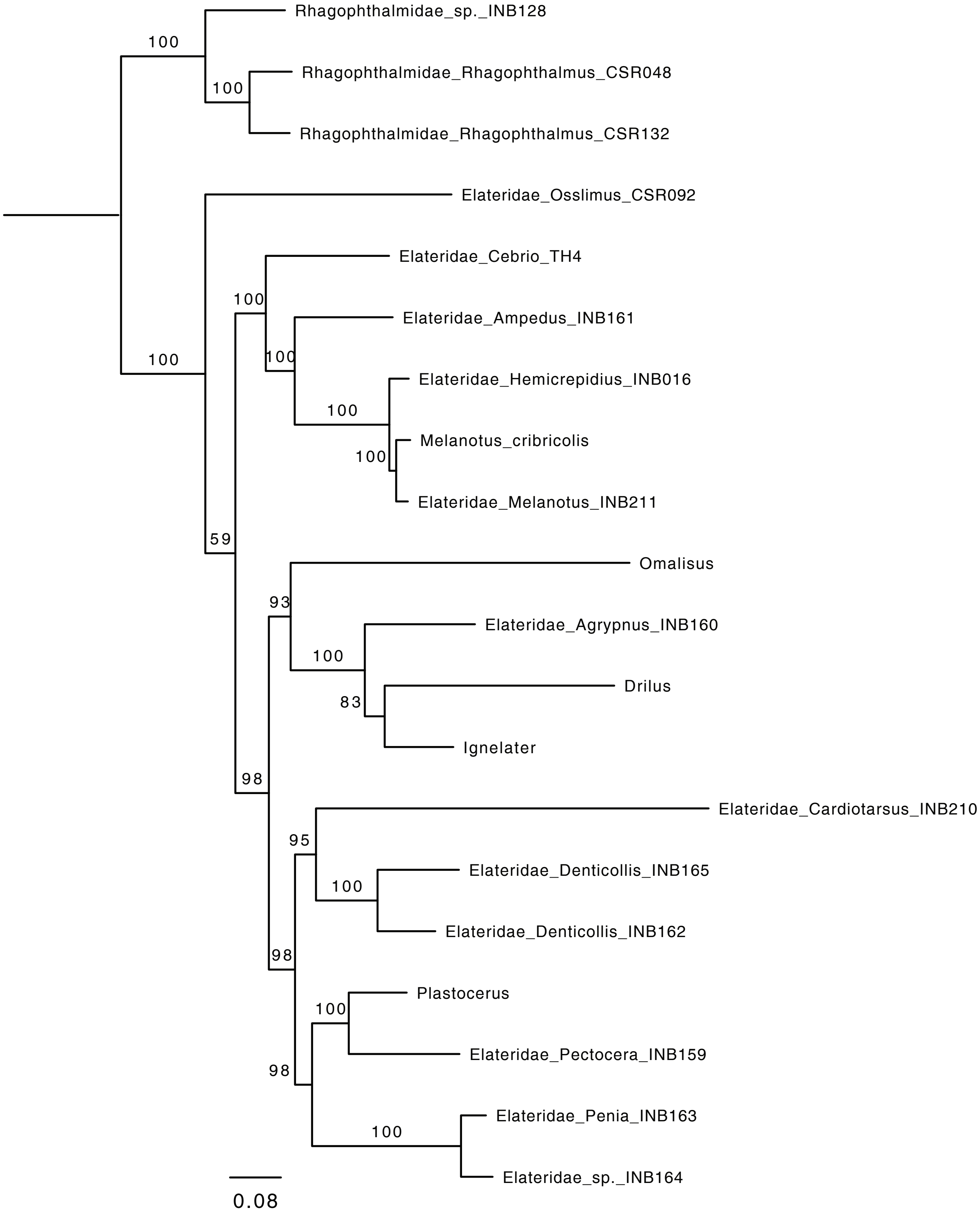

Supplementary figure S16. Maximum likelihood (IQ-Tree) topology recovered from 66-genes dataset at nucleotide level using only Elateridae taxa and Rhagophthalmidae as an outgroup.

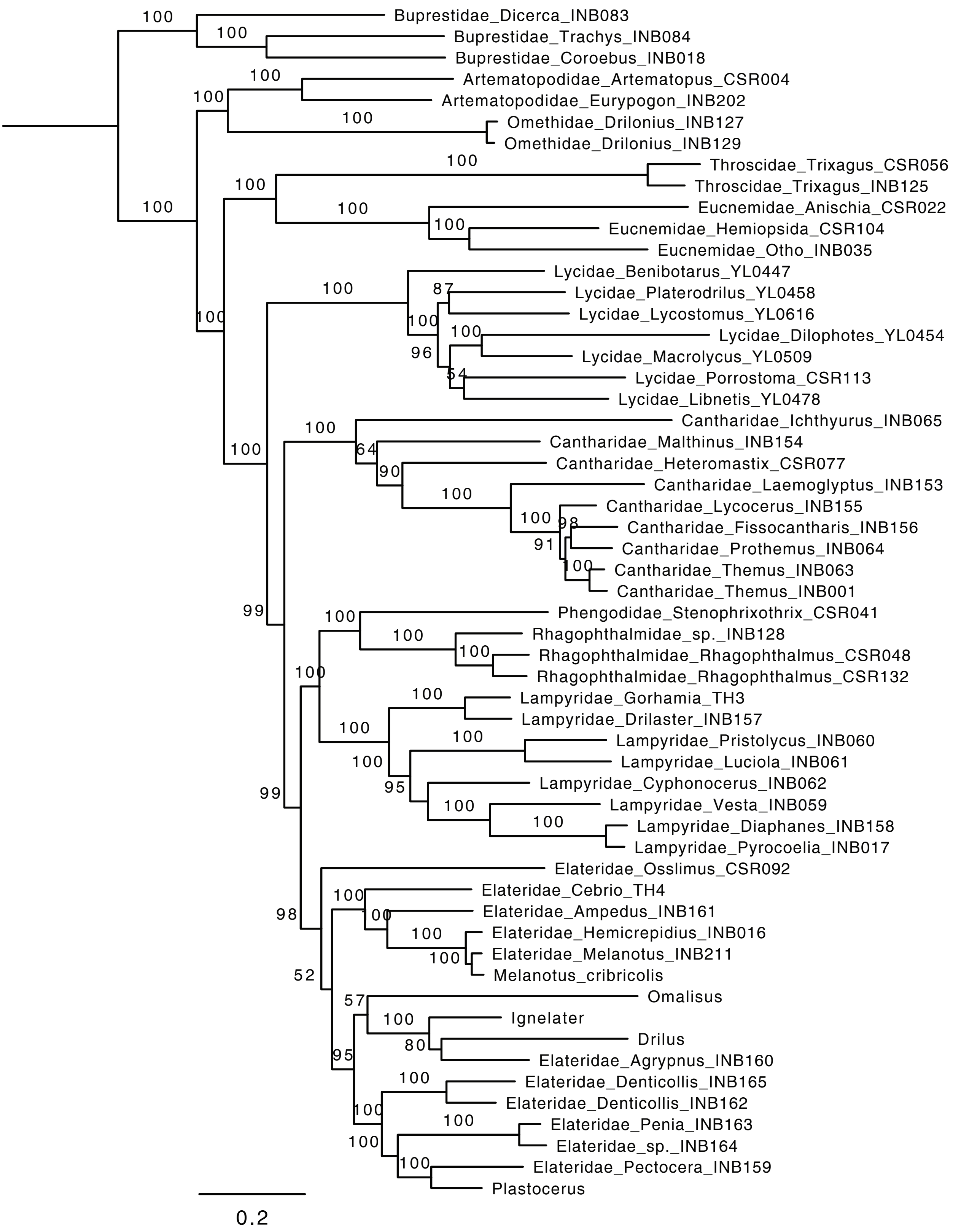

Supplementary figure S17. Maximum likelihood (IQ-Tree) topology recovered from 66-genes dataset at nucleotide level without Cardiotarsus (excluded due to high amount of missing data).

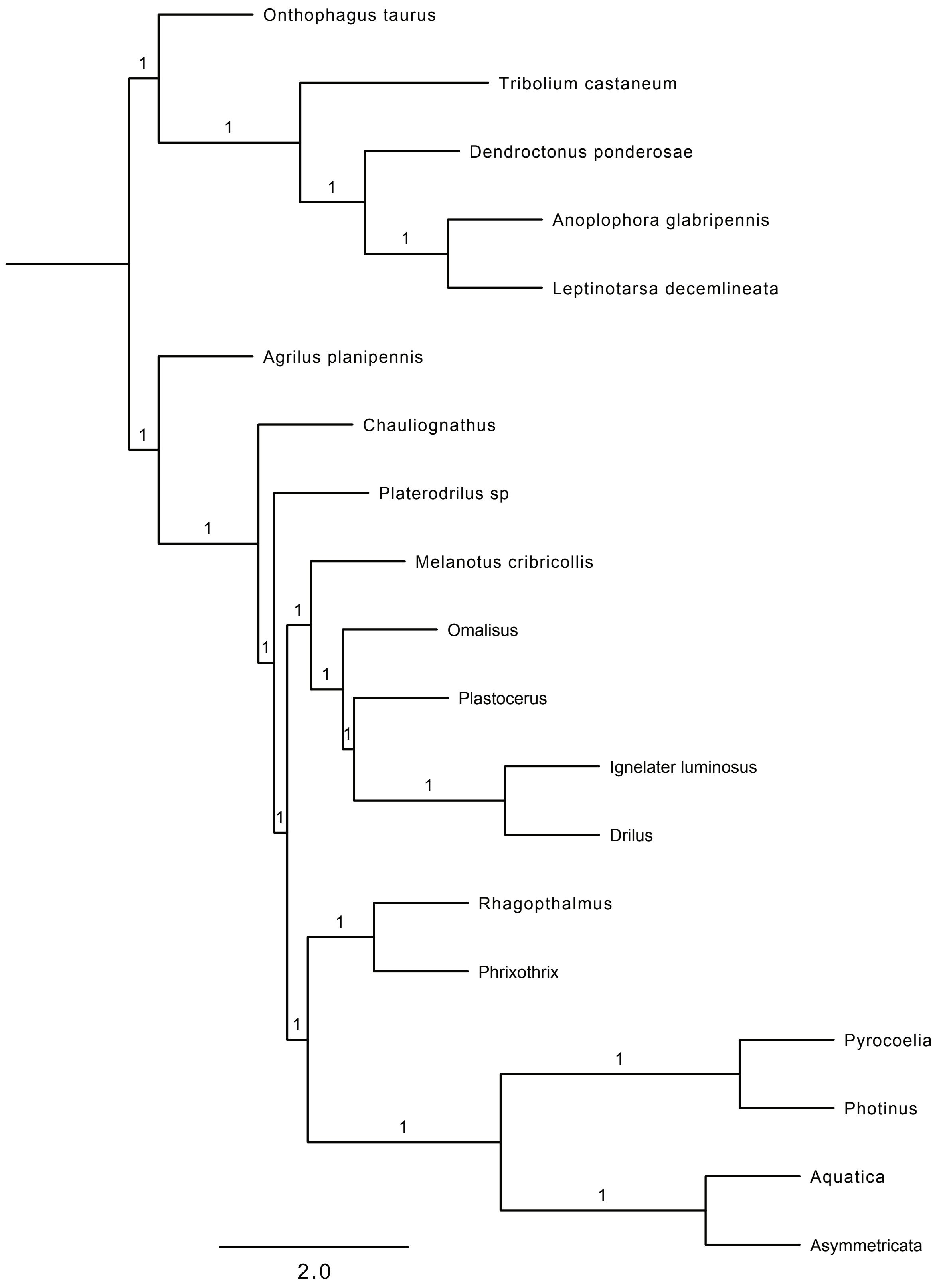

Supplementary figure S18. Coalescent species tree inferred by ASTRAL from 4202 single genes ML trees inferred by IQ-Tree at nucleotide level

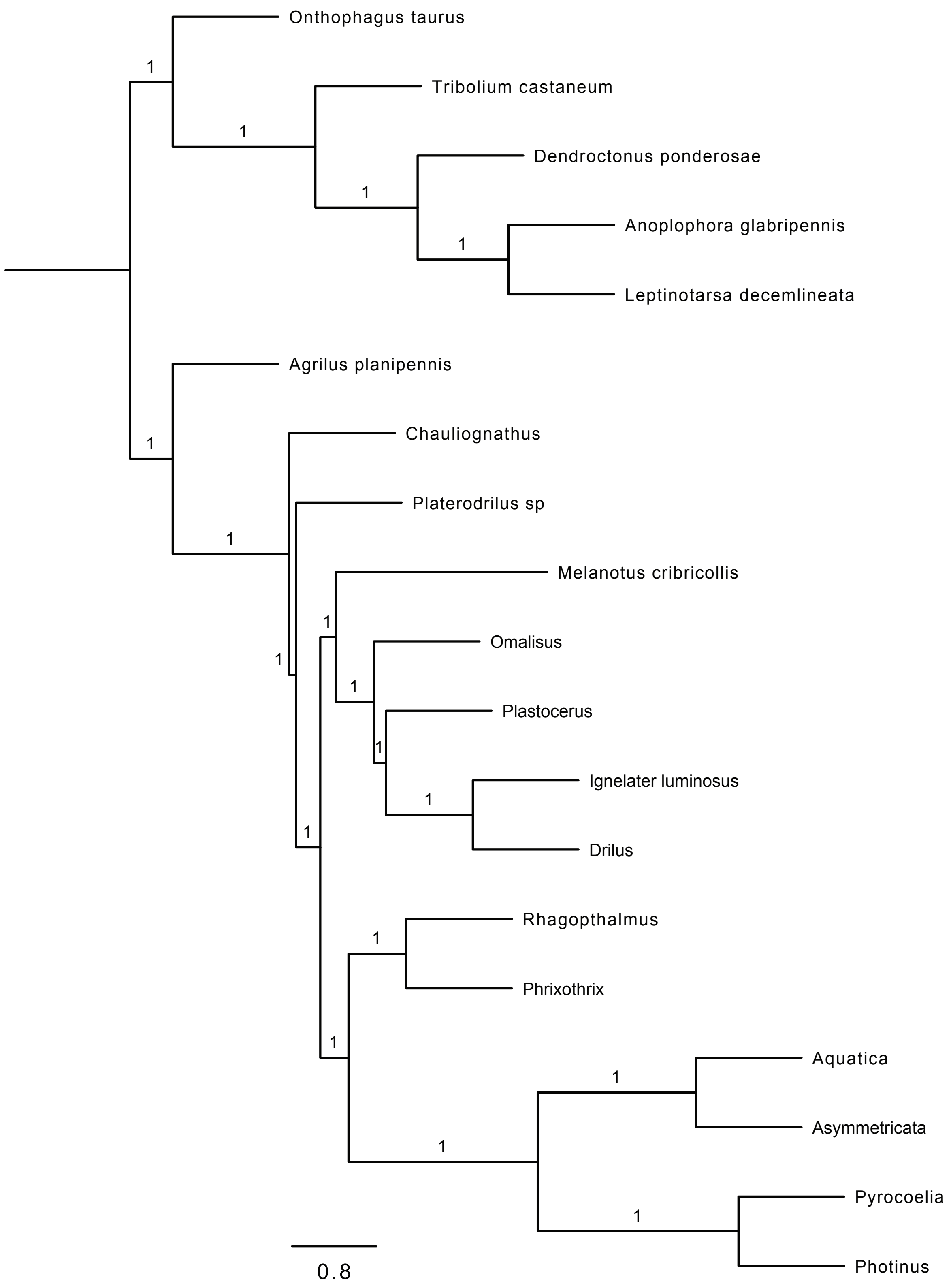

Supplementary figure S19. Coalescent species tree inferred by ASTRAL from 4202 single genes ML trees inferred by IQ-Tree at amino acid level

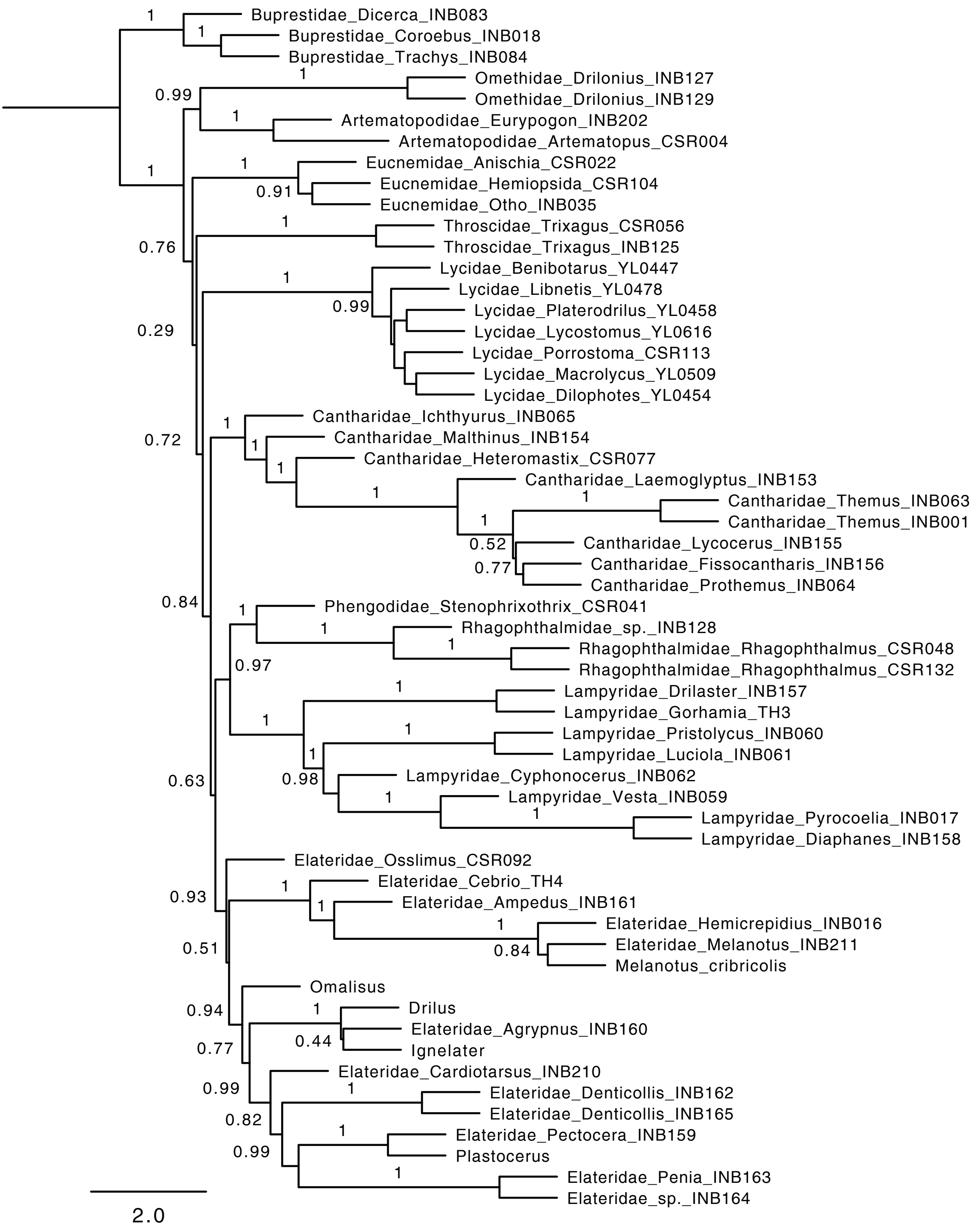

Supplementary figure S20. Coalescent species tree inferred by ASTRAL from 66 single genes ML trees inferred by IQ-Tree at nucleotide level.

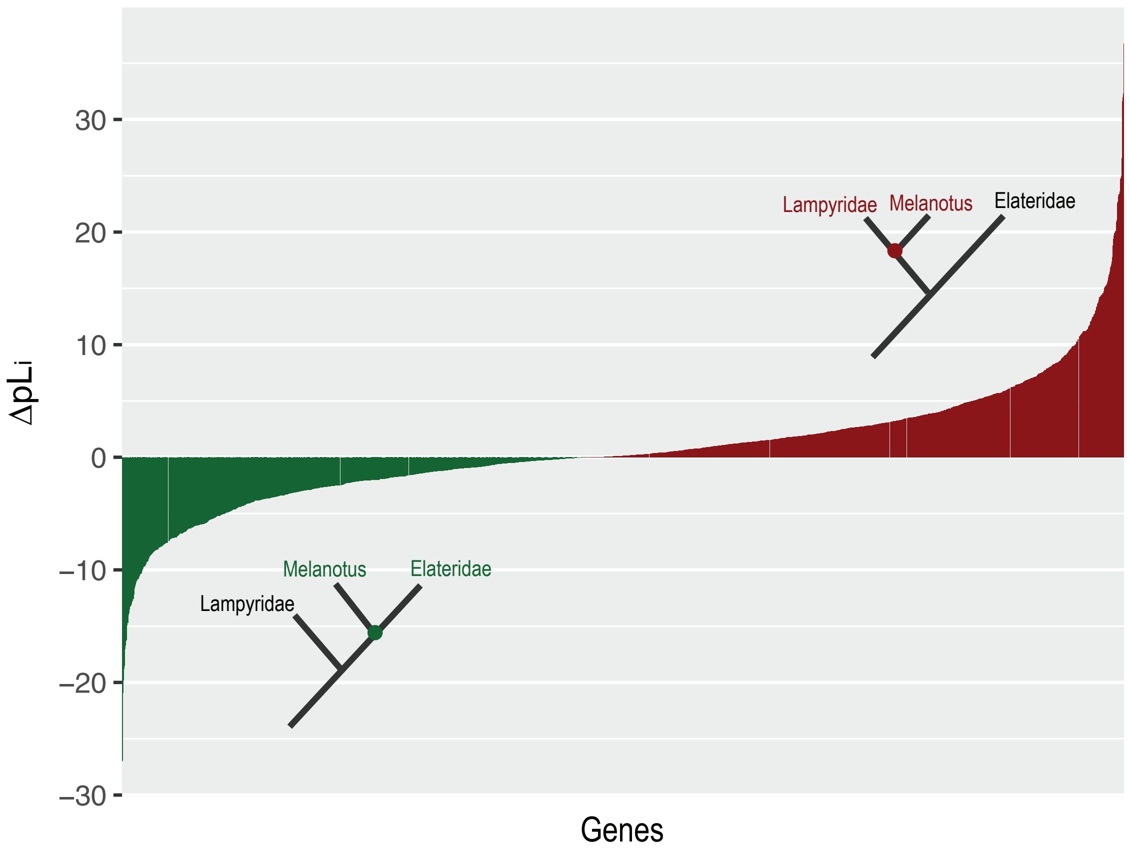

Supplementary figure S21. Calculated log-likelihood difference of each single gene partition scores at amino acid level received for (Melanotus(Rhagophthalmidae+,Lampyridae)) +(Elateridae) and alternative ((Rhagophthalmidae+Phengodidae)Lampyridae) +(Melanotus+Elateridae) topologies

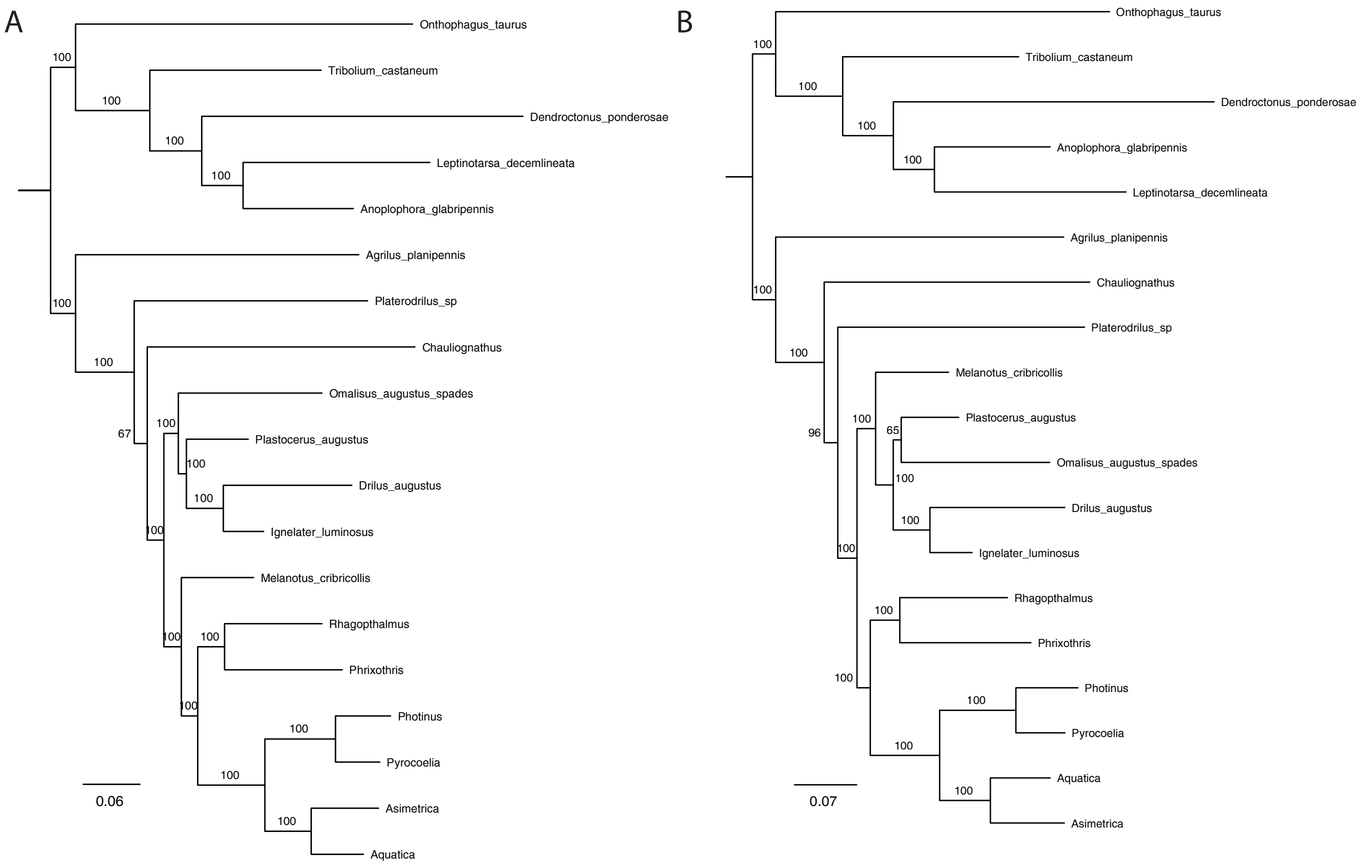

Supplementary figure S22. Maximum likelihood IQ-Trees topologies calculated at amino acid level which support (A) (Melanotus(Rhagophthalmidae+Lampyridae))+(Elateridae) 1098 genes or alternative (B) ((Rhagophthalmidae+Phengodidae)Lampyridae)+(Melanotus+ Elateridae) 959 genes.

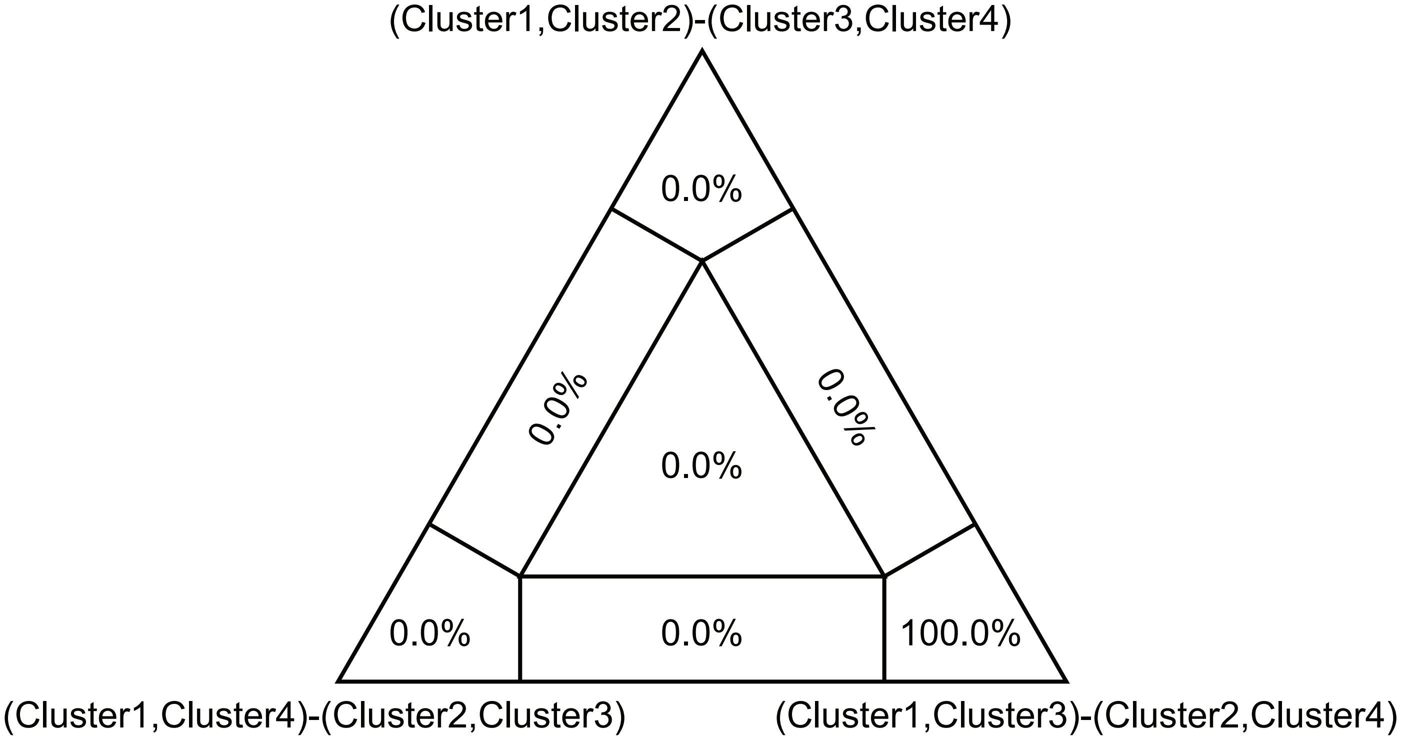

taxset Cluster1 = Phrixothrix, Rhagophthalmus, Aquatica, Asymmetrica, Photinus, Pyrocoelia
taxset Cluster2 = Omalisus
taxset Cluster3 = Melanotus
taxset Cluster4 = Drilus, Ignelater, Plastocerus

Supplementary figure S23. FcLM analysis of Omalisus position based on the amino acid matrix of 4202 filtered and partitioned orthologs.

A

	Omalisus v1 spades shovil
span (bp)	367,236,837
N (%)	0.13
GC (%)	34.55
AT (%)	65.45
scaffold count	379,187
longest scaffold (bp)	302,134
scaffold N50 length (bp)	9,222
scaffold N50 count	10,233
scaffold N90 length (bp)	223
scaffold N90 count	116,782
contig count	384,087
contig N50 length (bp)	7,889
contig N50 count	11,848
contig N90 length (bp)	223
contig N90 count	121,881

B

	Omalisus v1 megahit
span (bp)	355,482,086
N (%)	0.00
GC (%)	34.06
AT (%)	65.94
scaffold count	234,545
longest scaffold (bp)	255,042
scaffold N50 length (bp)	3,927
scaffold N50 count	22,848
scaffold N90 length (bp)	481
scaffold N90 count	126,658
contig count	234,545
contig N50 length (bp)	3,927
contig N50 count	22,848
contig N90 length (bp)	481
contig N90 count	126,658

C

	Plastocerus v1 megahit
span (bp)	463,025,604
N (%)	0.00
GC (%)	34.80
AT (%)	65.20
scaffold count	276,394
longest scaffold (bp)	84,730
scaffold N50 length (bp)	4,332
scaffold N50 count	26,464
scaffold N90 length (bp)	529
scaffold N90 count	153,458
contig count	276,394
contig N50 length (bp)	4,332
contig N50 count	26,464
contig N90 length (bp)	529
contig N90 count	153,458

D

	Drilus v1 megahit
span (bp)	495,090,403
N (%)	0.00
GC (%)	33.37
AT (%)	66.63
scaffold count	301,396
longest scaffold (bp)	49,253
scaffold N50 length (bp)	3,363
scaffold N50 count	40,128
scaffold N90 length (bp)	579
scaffold N90 count	177,393
contig count	301,396
contig N50 length (bp)	3,363
contig N50 count	40,128
contig N90 length (bp)	579
contig N90 count	177,393

Supplementary figure S24. Draft genomes assembly statistics for (A) *Omalisus* procesed with shovil pipeline and spades, (B) *Omalisus* assembly with megahit for comparison, (C) *Plastocerus* megahit assembly and (D) *Drilus* megahit assembly.
